# Supplementary material for: Designed folding pathway of modular coiled-coil-based proteins
Source: Nat Commun. 2021 Feb 11;12:940. doi: 10.1038/s41467-021-21185-5 (PMC7878764; doi:10.1038/s41467-021-21185-5)
Supplement: Supplementary file 1 — Supplementary Information [file 41467_2021_21185_MOESM1_ESM.pdf]

SUPPLEMENTARY INFORMATION

Designed folding pathway of modular coiled-coil-based proteins

Jana Aupič[1,#], Žiga Strmšek[1,2,#], Fabio Lapenta[1,3], David Pahovnik[4], Tomaž Pisanski[5], Igor Drobnak[1], Ajasja Ljubetič[1], Roman Jerala[1,3,*]

[1] Department of Synthetic Biology and Immunology, National Institute of Chemistry, Hajdrihova 19, SI-1000 Ljubljana, Slovenia

[2] Interdisciplinary Doctoral Programme in Biomedicine, University of Ljubljana, Kongresni trg 12, SI-1000 Ljubljana, Slovenia

[3] EN-FIST Centre of Excellence, Trg OF 13, SI-1000 Ljubljana, Slovenia

[4] Department of Polymer Chemistry and Technology, National Institute of Chemistry, Hajdrihova 19, SI-1000 Ljubljana, Slovenia

[5] FAMINT, University of Primorska, Glagoljaška 8, 6000 Koper, Slovenia and Institute of Mathematics, Physics and Mechanics, SI-1000 Ljubljana, Slovenia

[#] These authors contributed equally to this work

*Correspondence to: roman.jerala@ki.si

This PDF file includes:

Supplementary Discussion
Supplementary Figs. 1 to 18
Supplementary Tables 1 to 14

Supplementary Discussion

Thermal stability of individual CCs

Homodimers APHSN[1], BCRSN[2] and GCNSN[3] all contain a single polar residue in their hydrophobic interface. Additionally, in APHSN all e:g pairs can participate in the formation of salt bridges, resulting in its high melting temperature (> 80 ^{o}C). Both BCRSN and GCNSN exhibited lower thermal stability (T_m = 50 ^{o}C and 33 ^{o}C, respectively), due to a lower number of attractive e:g interactions and, in case of GCNSN, reduced peptide length. On the other hand, peptide pairs P3SN:P4SN, P5SN:P6SN and P7SN:P8SN (T_m = 33 ^{o}C, 28 ^{o}C and < 20 ^{o}C, respectively) all contain two energetically unfavourable Asn-Asn pairings in the hydrophobic interface, while all e:g interactions are complementary. Differences in their thermal stabilities can be explained by considering the residue pattern at heptad position a and the electrostatic pattern at e and g positions (in each heptad positions e and g are occupied by the same residue, Supplementary Table 1). It has been previously shown that positioning the Asn residues at the a site in the third and fourth heptad leads to CC assemblies with higher thermodynamic stability (IINN > NINI ≈ ININ)[4,5]. Additionally, CCs with the same charges in neighbouring heptads demonstrated higher melting temperatures (EEKK > EKKE > EKEK)[5].

Definition of intra-chain distance between peptide modules

Spatial proximity of pairing peptide segments in the unfolded polypeptide chain and in the partially folded intermediates was described using the intra-chain distance metric. We defined the intra-chain distance as the minimal number of peptide segments between matching peptide segments. For each module, two distances are calculated (Supplementary Fig. 1). For antiparallel CCs, such as APHSN (red arrows in Supplementary Fig. 1), the distance is calculated between the opposing termini (N1-C2 and C1-N2; N1 signifies the N-terminus of the 1st peptide partner and C2 the C-terminus of the 2nd partner), while for parallel CCs, such

as P3SN:P4SN (blue arrows in Supplementary Fig. 1), matching termini are used for distance calculation (N1-N2 and C1-C2). In both cases, the minimum of the two values is taken as the intra-chain distance. For example, in Supplementary Fig. 1a the distance C1-N2 would be taken as the intra-chain distance for the APHSN module. As the peptide segments pair up, the effective intra-chain distance may decrease. In case matching peptide segments are connected by multiple paths, the number of segments separating the pairing termini is calculated for all paths, and the shortest path is taken as the intra-chain distance. For example, in Supplementary Fig. 1b two paths lead from peptide P3SN to its partner P4SN. The number of segments between pairing termini is the lowest along the path 1 (assembled CCs are counted as one segment). The N1-N2 distance along the path 1 is therefore taken as the intra-chain distance.

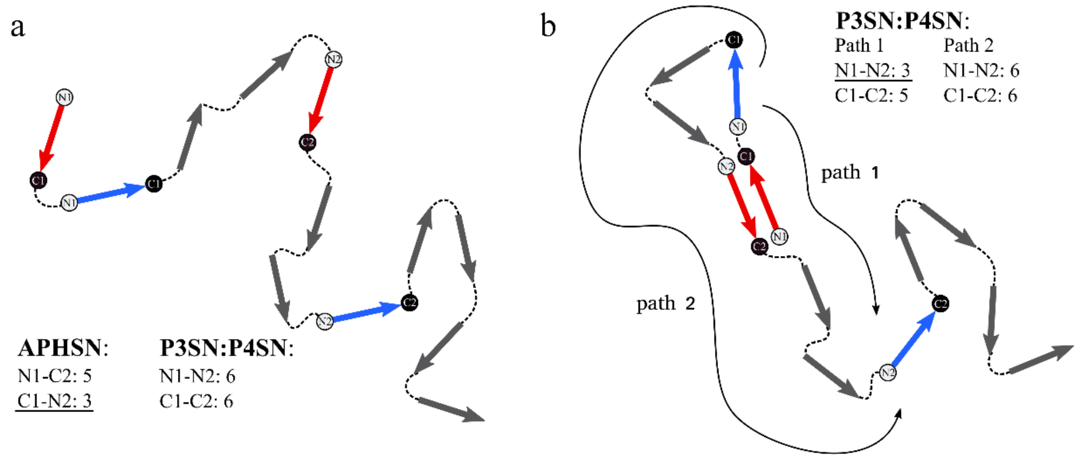

Supplementary Figure 1. Examples of intra-chain distance calculation for two CC modules, antiparallel APHSN (red) and parallel P3SN:P4SN (blue), in the unfolded state (a) and after one segment has been paired (b).

Folding kinetics of the APHSN module in TET12SN

To characterize the folding mechanism of the APHSN module in TET12SN, a pair of cysteine residues for fluorescent dye labelling was introduced in its central heptad. The observed folding constant k_{obs} in 1 M Gdn-HCl decreased in comparison to k_{obs} determined in

2 M Gdn-HCl, resulting in the so-called roll-over effect (Supplementary Fig. 9a). The analysis of refolding amplitudes revealed they were lower than expected from the equilibrium stability measurements, indicating a “burst-phase” occurred in the instrumental dead-time, τ_d (Supplementary Fig. 9b). This can be the result of either a three-state folding mechanism with a marginally stable intermediate rapidly forming before the major kinetic barrier or of very fast folding, which results in only a fraction of the refolding process being detected leading to an underestimation of the folding constant[6]. We posit the roll-over effect observed for the APHSN module is due to its rapid folding for several reasons:

1. Reducing τ_d from 4 ms to 2 ms led to an increase in k_{obs} from 31 to 53 s^{-1} (Supplementary Fig. 9c).
2. Previously published global folding experiments[7], where fluorescent probes were placed at the C- and N-terminal end of TET12SN were fit in the range between 0 and 0.3 s. The refolding constant in 1 M Gdn-HCl was 40 s^{-1} (Supplementary Fig. 10). Consequently, no cage edge should display k_{obs} below that value. Conversely, the global refolding constant is in good agreement with k_{obs} for the P5SN:P6SN edge, which is the last to fold if we exclude the APHSN module.
3. Rapid refolding has been previously observed for small proteins such as mammalian ubiquitin[8] and the KIX domain[9] composed of three interacting α-helices. Their refolding has been initially misidentified to proceed according to a three-state mechanism[10,11]. Only use of special instrumentation with $\tau_d < 1.3$ ms revealed their folding proceeds according to a two-state mechanism with k_{obs} approaching 1000 s^{-1} in the absence of a denaturant. The high thermodynamics stability and helical propensity[12] of the APHSN module (71 %[13]) is another tacit indication that the APHSN module could experience rapid refolding.

Supplementary Figures

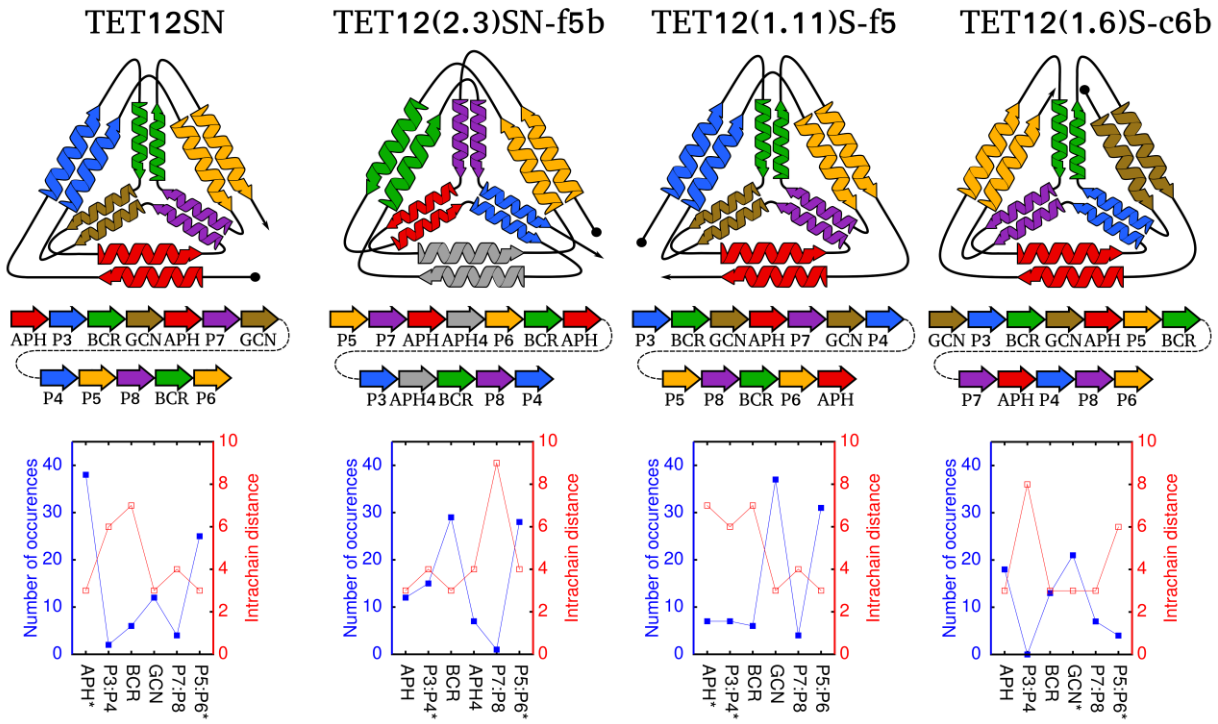

Supplementary Figure 2. Coiled-coil (CC) forming pairs positioned at shorter intra-chain distances in the polypeptide chain display faster folding in Gō molecular dynamics (MD) simulations. Schemes of CCPO tetrahedra whose folding has been investigated by Gō simulations are shown in the upper panel. A 100 MD runs were performed for each tetrahedron variant with each run consisting of 30×10^6 steps. Corresponding graphs in the lower panel depict the correlation between CC module's intra-chain distance and the number of folding runs where the pair was observed to be first to fold. A CC pair was considered folded after 50 % of its native contacts were formed. Terminally positioned CC modules, denoted with an asterisk, were observed to form first more frequently than expected based on their intra-chain distance. For example, while APH4 and P5:P6 share the same intra-chain distance in TET12(2.3)SN-f5b, terminally positioned pair P5SN:P6SN is first to fold in 28 MD runs, while APH4 assembles first only in 7 out of a total of 100 MD runs. Source data are provided as a Source Data file.

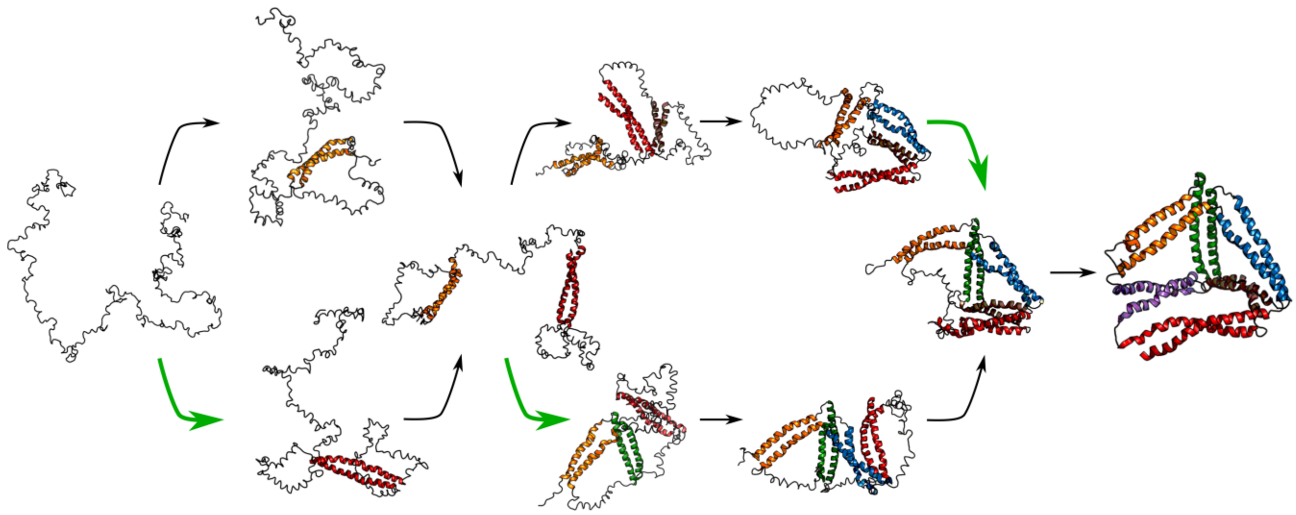

Supplementary Figure 3. The most frequently observed folding steps in Gō simulations for TET12SN. Folding transitions exhibiting the highest flux are denoted by green arrows. Experimentally determined ensemble kinetics indicated folding proceeds from the N- to the C- terminal end which has not been recapitulated by molecular dynamics simulations.

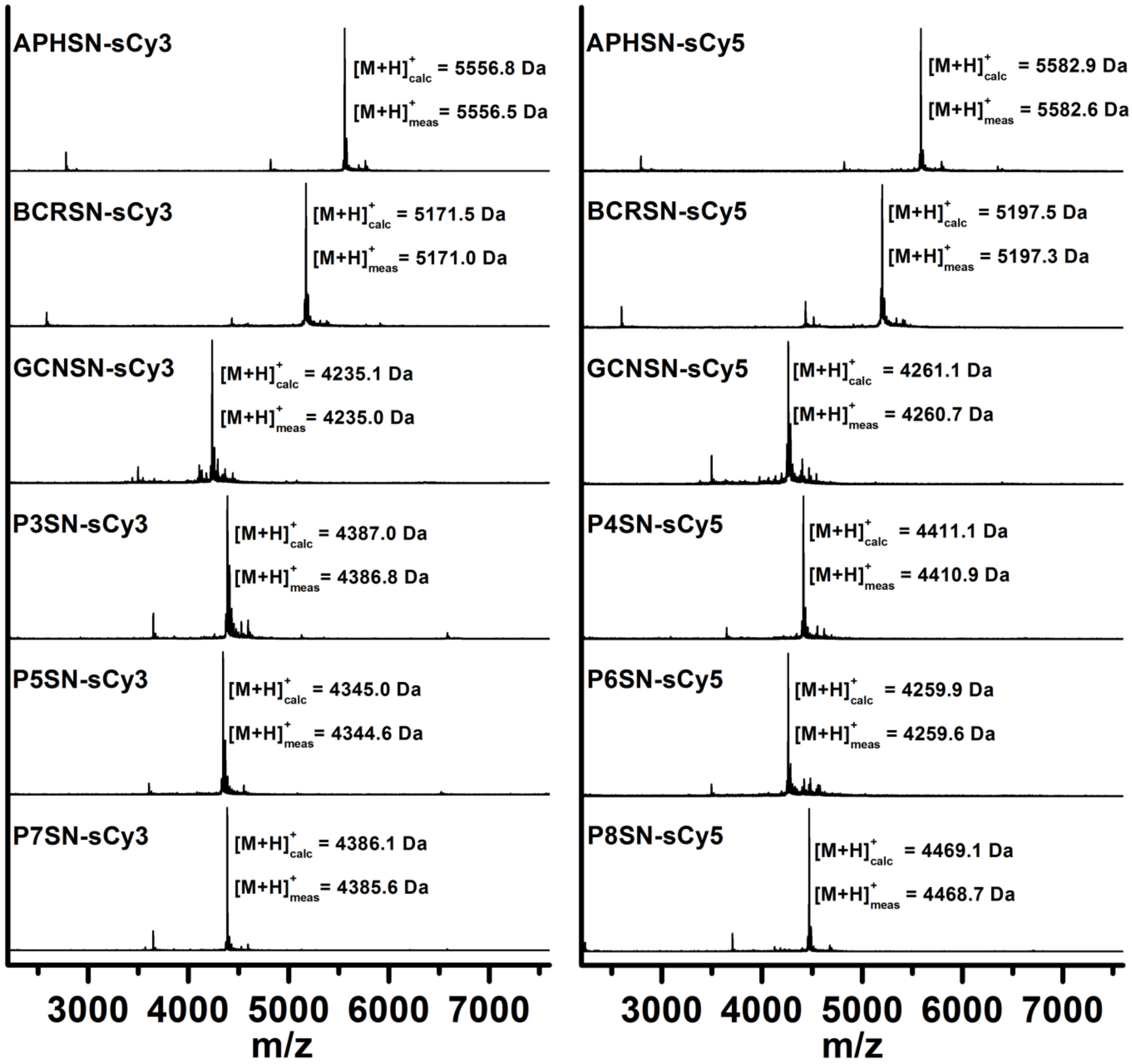

Supplementary Figure 4. MALDI-TOF spectra of purified fluorescently labelled peptides with sCy3 and sCy5. Measured monoisotopic signals are in good agreement with the calculated exact masses ionized with the proton. Source data are provided as a Source Data file.

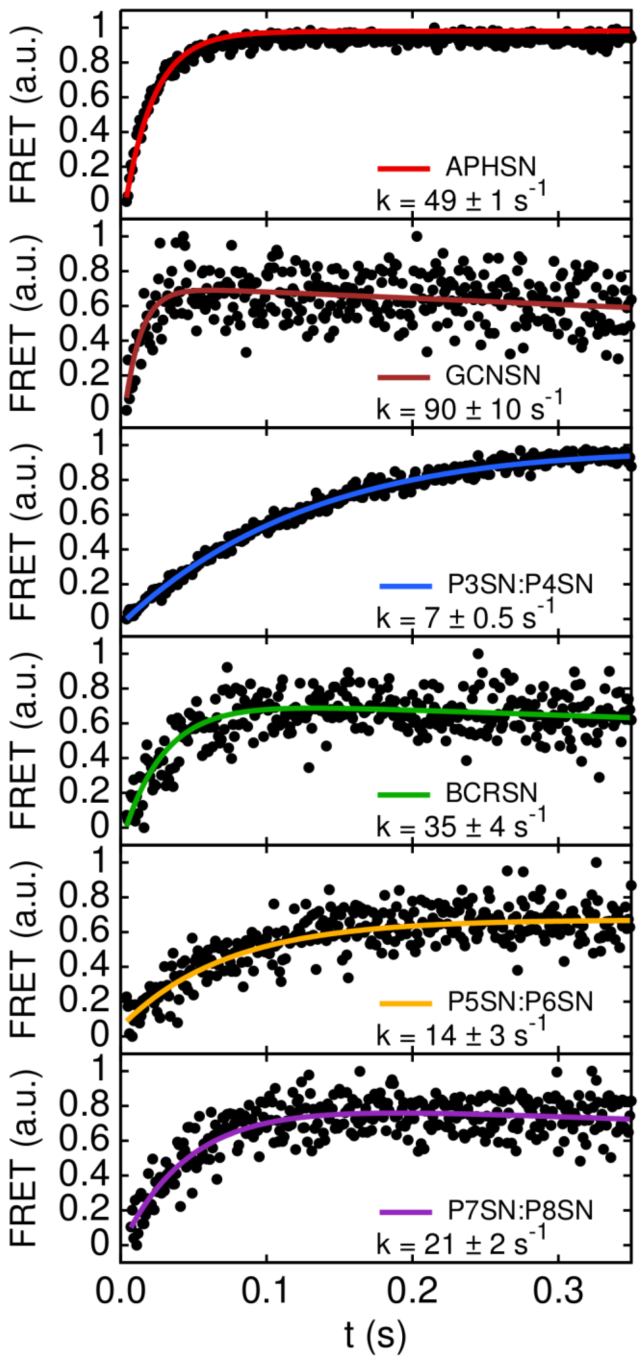

Supplementary Figure 5. FRET stopped-flow traces showing refolding kinetics for individual CC dimers at 20 μM dimer concentration. CC pairs were refolded in 1 M Gdn-HCl. Source data are provided as a Source Data file.

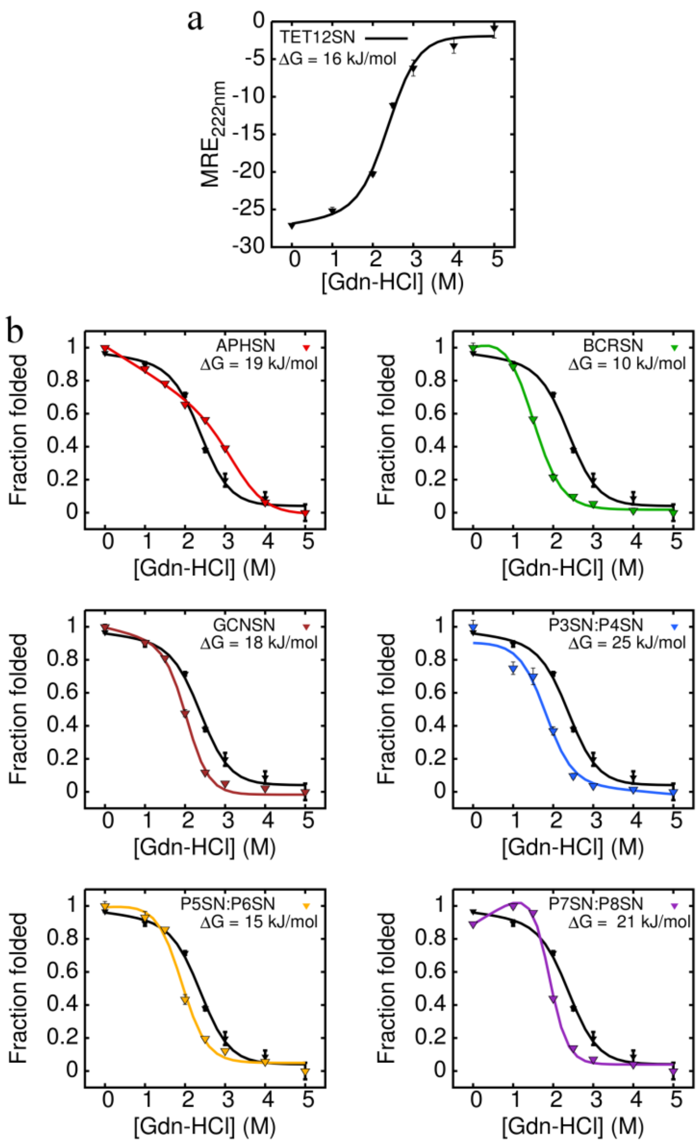

Supplementary Figure 6. Chemical denaturation profiles for TET12SN. (a) Secondary structure content of TET12SN as a function of Gdn-HCl concentration was monitored by circular dichroism. Data are presented as mean values +/- SD (n = 3 technical repetitions). (b) Unfolding of individual CC edges in TET12SN was tracked by observing the FRET signal as a function of Gdn-HCl concentration. Data are presented as mean values +/- SD (n = 3 independent samples). In each graph CD denaturation profile is shown in black for comparison. In all cases, experimental data (symbols) were fit with a two-state model (curves). While CD denaturation profile suggests unfolding proceeds as a cooperative two-state mechanism, FRET measurements indicate that all CC pairs do not unfold in concert. Source data are provided as a Source Data file.

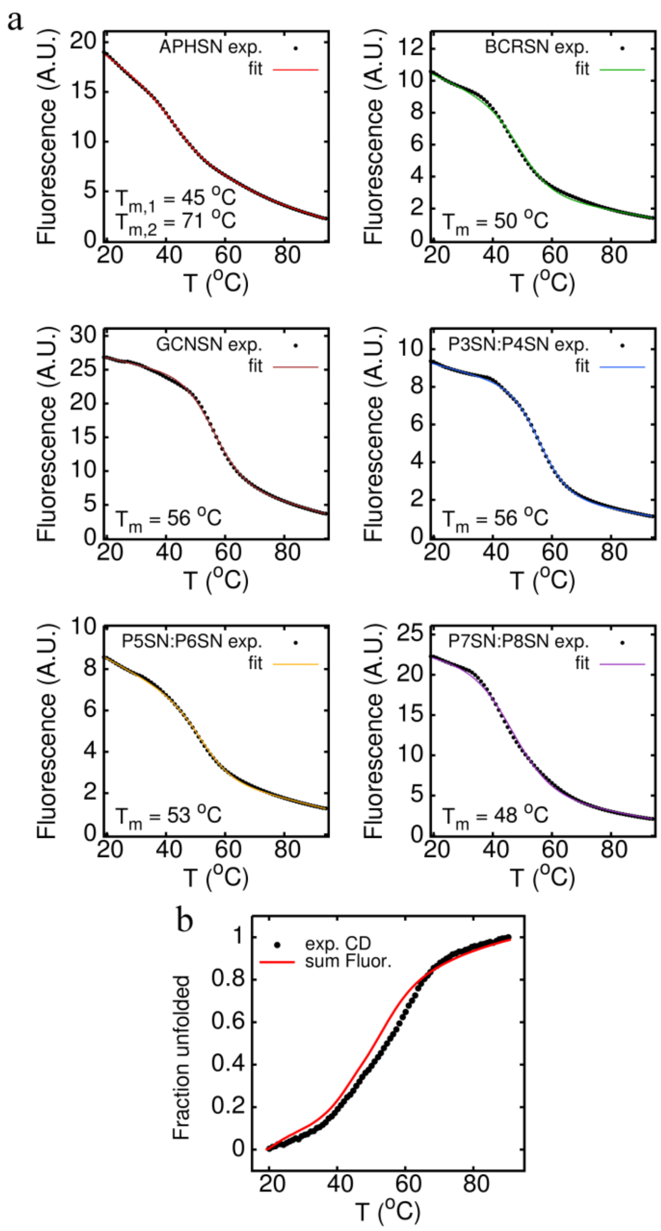

Supplementary Figure 7. Thermal unfolding of fluorescently labelled cage edges in TET12SN. (a) The dependence of acceptor fluorescence as a function of temperature for different positions of the fluorescent dye pair. Melting temperatures were determined by fitting a two-state thermodynamic model (solid curve) to experimental data (black dots), except for the APHSN module which exhibited a weak second transition at elevated temperatures. (b) The fluorescent traces were normalized, averaged (red curve) and compared to the normalized thermal denaturation profile of TET12SN as observed by circular dichroism (black dots). Source data are provided as a Source Data file.

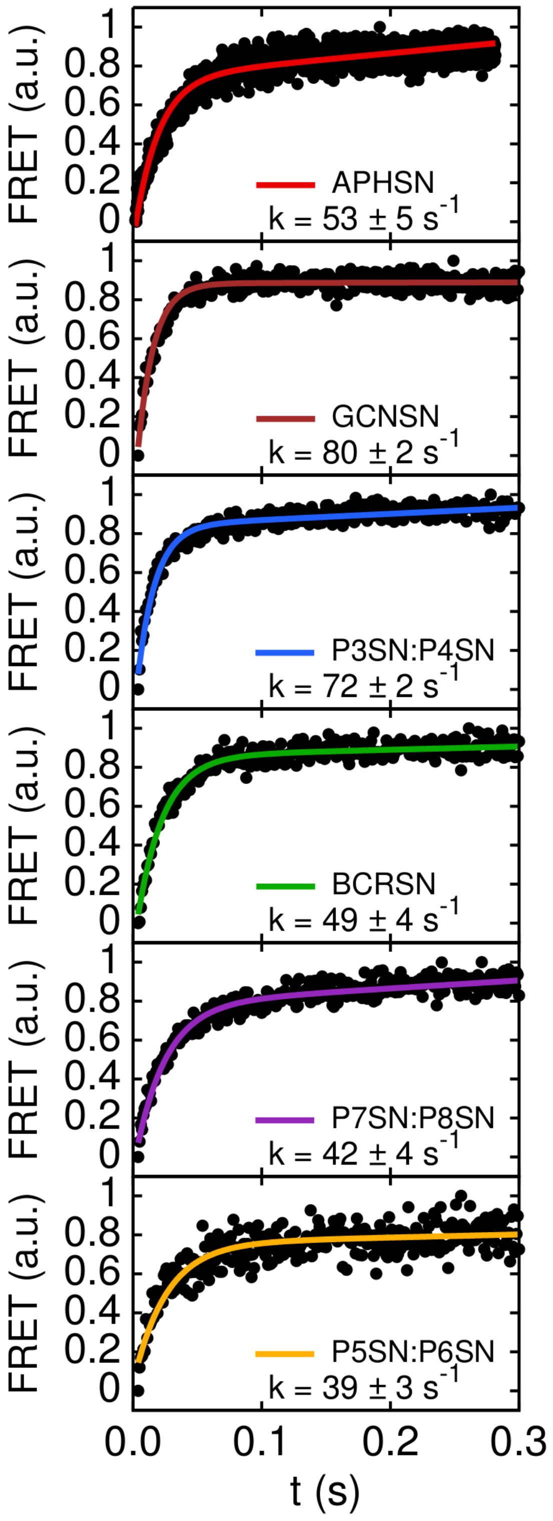

Supplementary Figure 8. FRET stopped-flow traces showing refolding kinetics for individual CC edges in TET12SN in 1 M Gdn-HCl. Source data are provided as a Source Data file.

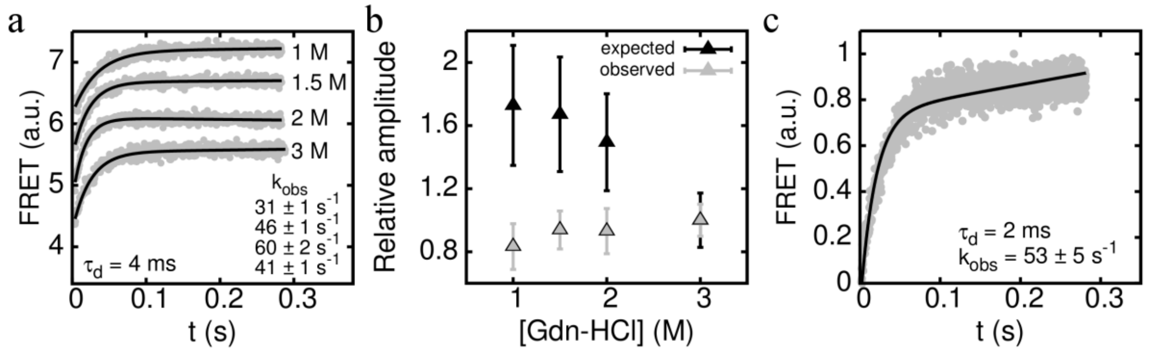

Supplementary Figure 9. Folding kinetics of the APHSN module in TET12SN. (a) To track the folding of the APHSN module a cysteine mutation was introduced into the central heptad of both APHSN peptide segments. For labelling, the Sulfo-cy3/Sulfo-cy5 dye pair was utilized. The protein was unfolded in 5 M Gdn-HCl and refolded at different Gdn-HCl concentrations. The observed folding constant (k_{obs}, listed in order of increasing Gdn-HCl concentration) decreased as Gdn-HCl concentration was lowered. (b) The observed refolding amplitudes (grey symbols, mean +/- SD, n ≥ 4) were lower than expected from equilibrium measurements (black symbols, mean +/- SD, n = 3), indicating a burst phase in the dead-time of the stopped-flow instrument. (c) Decreasing the dead time from 4 ms to 2 ms led to an increase in the observed folding constant. This indicates the observed roll-over is most likely due to technical limitations of the utilized stopped-flow apparatus and not due to the presence of a partially folded kinetic intermediate. Source data are provided as a Source Data file.

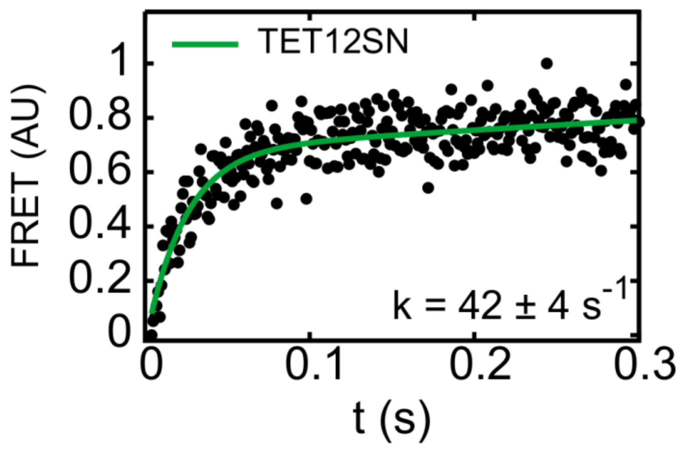

Supplementary Figure 10. Kinetics of global refolding for TET12SN in 1 M Gdn-HCl. Fluorescent probes were placed at the C- and N-terminal that come in close proximity as the tetrahedral cage folds. Source data are provided as a Source Data file.

a

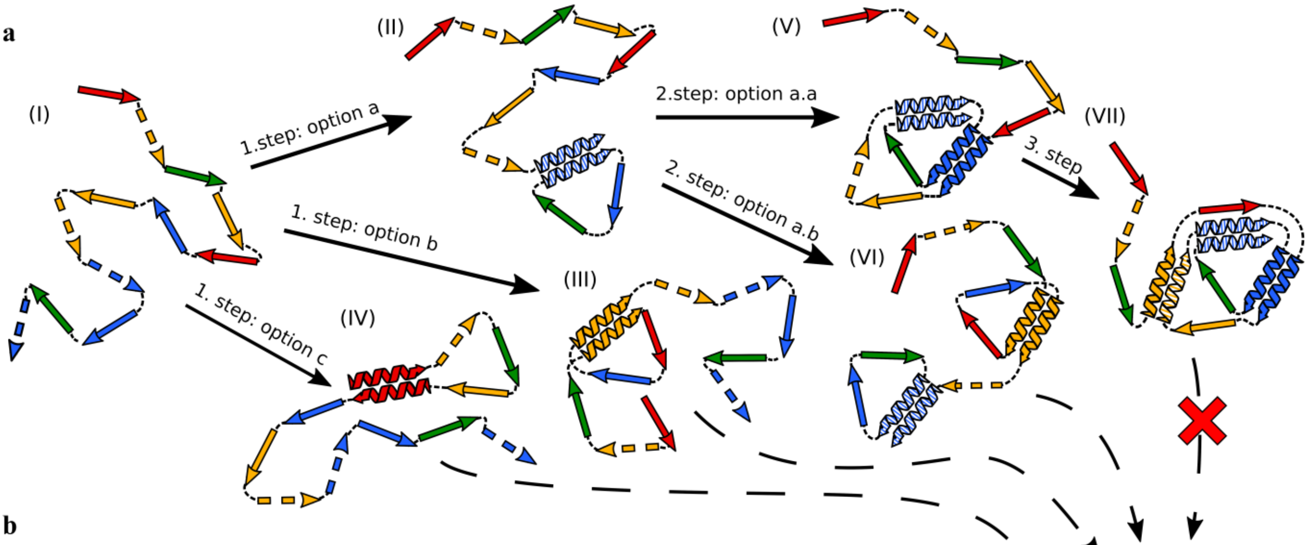

b

Arrangement of peptide building blocks in the polypeptide sequence	P_F
APHshSN-P5SN-BCRSN-P3SN-APHshSN-P3SN-P4SN-P6SN-P5SN-P4SN-BCRSN-P6SN	100
APHshSN-P6SN-BCRSN-P3SN-APHshSN-P3SN-P4SN-P5SN-P5SN-P4SN-BCRSN-P6SN	96.72
APHshSN-P3SN-BCRSN-P3SN-APHshSN-P5SN-P4SN-P4SN-P5SN-P6SN-BCRSN-P6SN	90.32
APHshSN-P3SN-BCRSN-P5SN-APHshSN-P3SN-P6SN-P4SN-P5SN-P4SN-BCRSN-P6SN	0
APHshSN-P3SN-BCRSN-P5SN-APHshSN-P4SN-P6SN-P4SN-P5SN-P3SN-BCRSN-P6SN	0
APHshSN-P3SN-BCRSN-P3SN-APHshSN-P6SN-P4SN-P4SN-P5SN-P5SN-BCRSN-P6SN	0
APHshSN-P3SN-BCRSN-P4SN-APHshSN-P6SN-P3SN-P4SN-P5SN-P5SN-BCRSN-P6SN	0
APHshSN-P6SN-BCRSN-P4SN-APHshSN-P3SN-P3SN-P5SN-P5SN-P4SN-BCRSN-P6SN	0
APHshSN-P5SN-BCRSN-P4SN-APHshSN-P3SN-P3SN-P6SN-P5SN-P4SN-BCRSN-P6SN	0
APHshSN-P3SN-BCRSN-P6SN-APHshSN-P4SN-P5SN-P4SN-P5SN-P3SN-BCRSN-P6SN	0
APHshSN-P3SN-BCRSN-P4SN-APHshSN-P5SN-P3SN-P4SN-P5SN-P6SN-BCRSN-P6SN	0
APHshSN-P3SN-BCRSN-P6SN-APHshSN-P3SN-P5SN-P4SN-P5SN-P4SN-BCRSN-P6SN	0

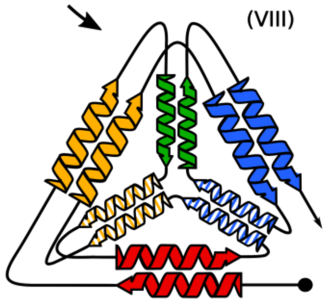

Supplementary Figure 11. Scheme of the CCPO folding model applied to the design of CCPO tetrahedra comprising repeats of coiled-coil modules. (a) Schematic representation of folding of a polypeptide chain with two instances of P5SN:P6SN (yellow arrows) and P3SN:P4SN (blue arrows) into a CCPO tetrahedron according to the proposed intra-chain distance model. Folding proceeds in discrete steps and is determined by the effective intra-chain distance between interacting coiled-coil pairs. To differentiate between productive and unproductive pairings the first pair copy is shown with solid arrows, while the second pair is represented with dotted lines or colouring. (b) Probability of folding (P_F) was calculated for all possible arrangements of peptide building blocks in order to identify the optimal arrangement of repeating coiled-coil pairs in the sequence. The table shows results of folding simulations for different polypeptide sequences based on the circular permutation 1.10 and containing two instances of coiled-coil pairs P5SN:P6SN and P3SN:P4SN, with P_F reflecting the percentage of successful assembly simulations.

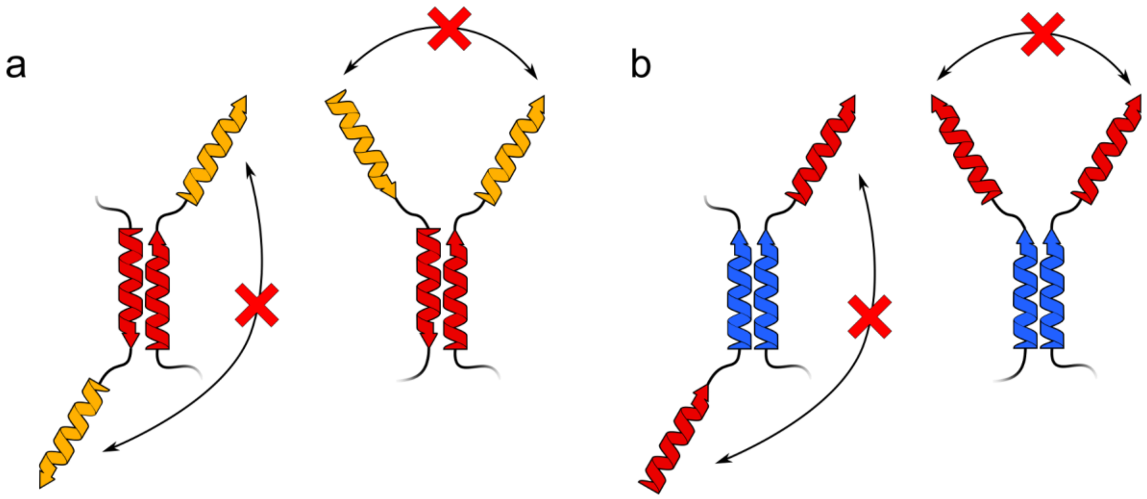

Supplementary Figure 12. Deterministic folding model presupposes peptides are rigid bodies prohibiting certain folding events due to sterical hindrances. (a) Forbidden pairings for parallel coiled-coils. (b) Forbidden pairings for antiparallel coiled-coils.

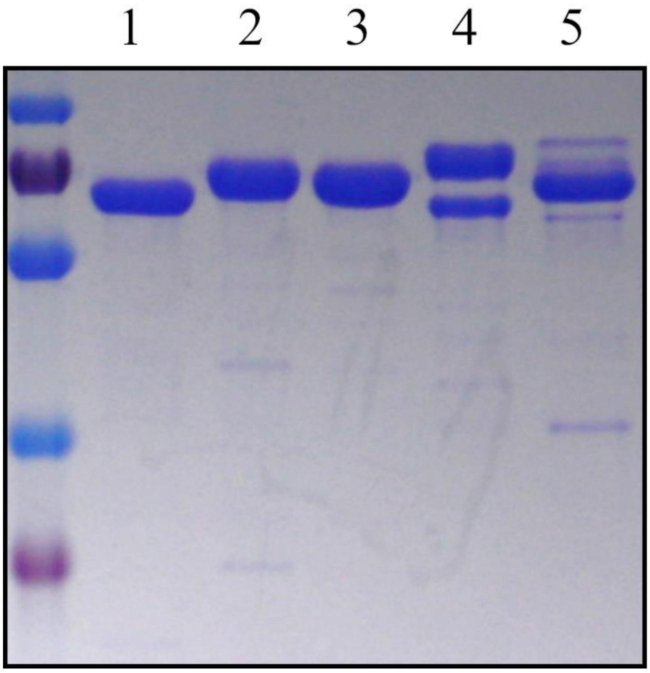

Supplementary Figure 13. SDS pages of purified CCPO tetrahedrons next to molecular markers (from top to bottom: 100 kDa, 70 kDa, 55 kDa, 35 kDa, 25 kDa). The lanes correspond to (1) TET12SN(2CC), (2) TET12SN(22CC), (3) TET12SN(222CC), (4) TET12SN(3CC), and (5) TET12SN(3CC)-neg. SDS-PAGE experiments were repeated at least three times for each protein with comparable outcomes. Uncropped gel is provided as a Source Data file.

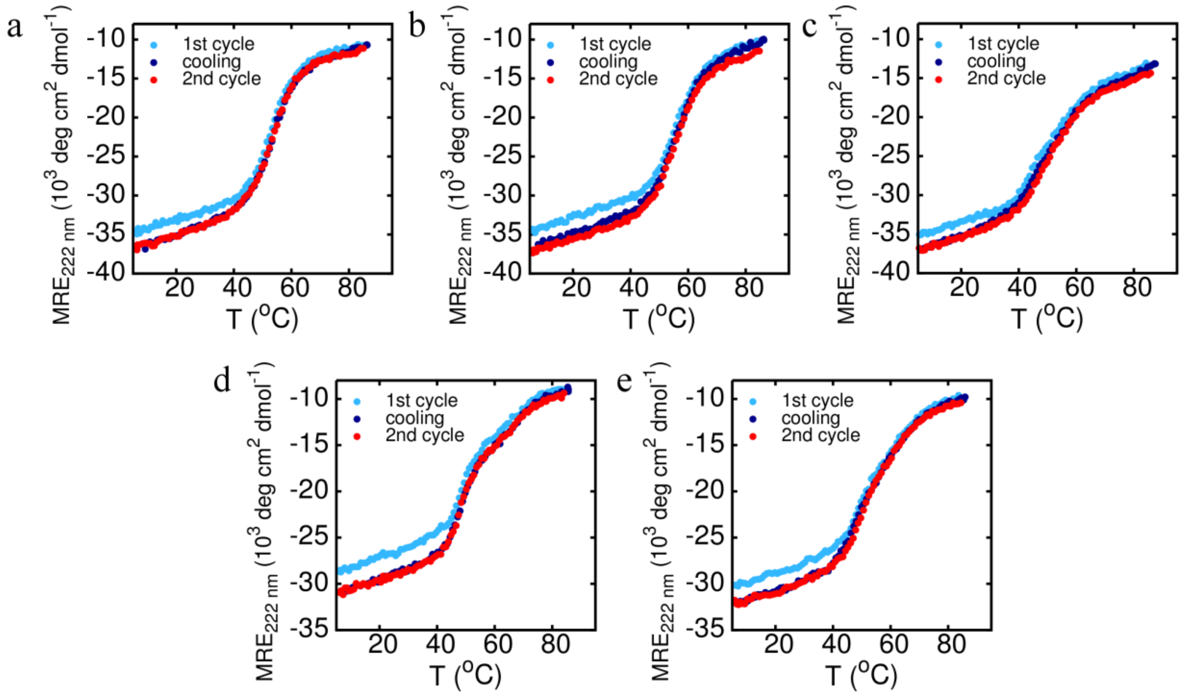

Supplementary Figure 14. Mean residue ellipticity (MRE) at 222 nm as a function of temperature for (a) TET12SN(2CC), (b) TET12SN(22CC), (c) TET12SN(222CC), (d) TET12SN(3CC) and (e) TET12SN(3CC)-neg. Good agreement of thermal unfolding (light blue dots) and refolding profiles (dark blue dots) suggests thermal unfolding is a thermodynamically reversible process. After refolding, denaturation profiles ware measured again (red dots). Source data are provided as a Source Data file.

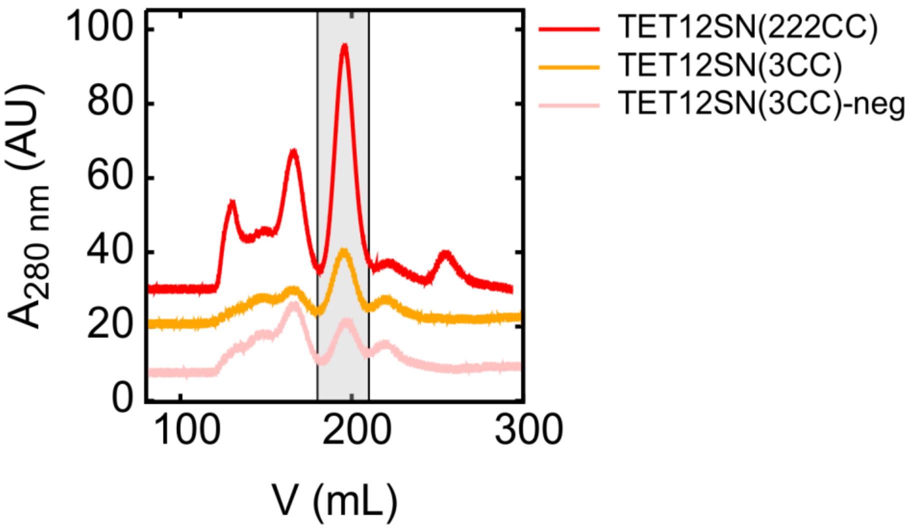

Supplementary Figure 15. Size exclusion chromatography (SEC) profiles after a Ni-NTA step for TET12SN(222CC), TET12SN(3CC) and TET12SN(3CC)-neg. While all proteins could be isolated in their monomeric form (grey shaded area), the yield for TET12SN(3CC) and TET12SN(3CC)-neg was lower. Source data are provided as a Source Data file.

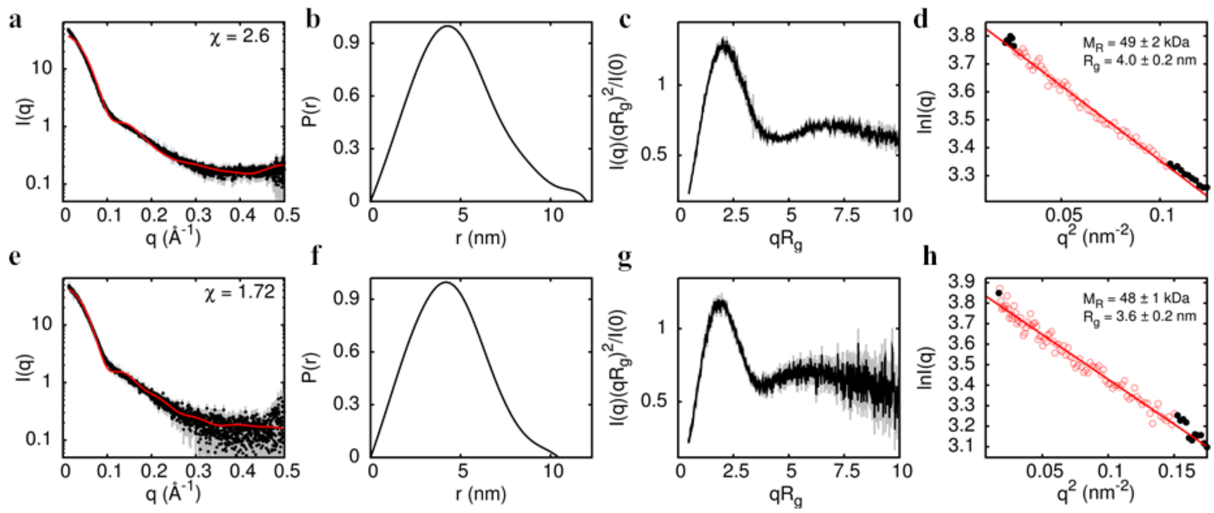

Supplementary Figure 16. SAXS analysis of CCPO tetrahedrons with three repeats of the P5SN:P6SN module. Upper panels show (a) comparison between theoretical (red line) and measured (black dots) scattering, (b) pair-distance distribution function, (c) Kratky plot, and (d) Guinier plot for TET12SN(3CC), while corresponding graphs in the lower panels (e-h) display equivalent plots for TET12SN(3CC)-neg. Experimental error is shown in grey (panels a, c and e, g). Data in panels a, c and e, g are presented as mean +/- SD (n = 40 technical repetitions). Source data are provided as a Source Data file.

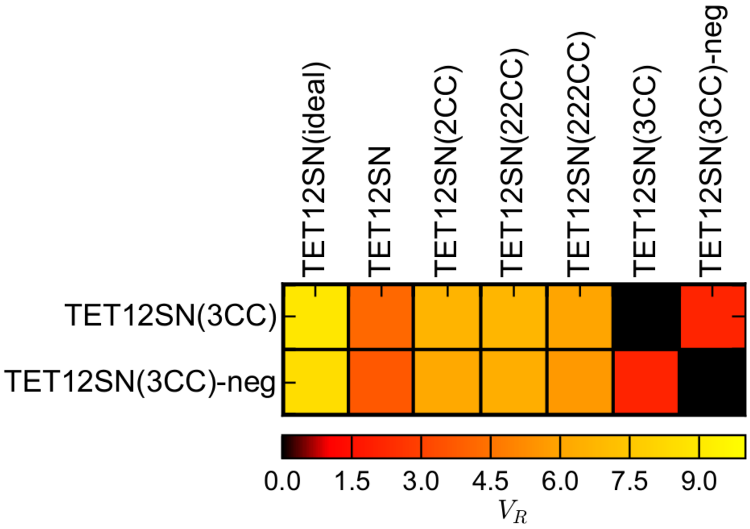

Supplementary Figure 17. SAXS similarity matrix comparing the scattering profiles of TET12SN(3CC) and TET12SN(3CC)-neg to those observed for other tetrahedron designs and to the scattering calculated for an ideal tetrahedral cage. Source data are provided as a Source Data file.

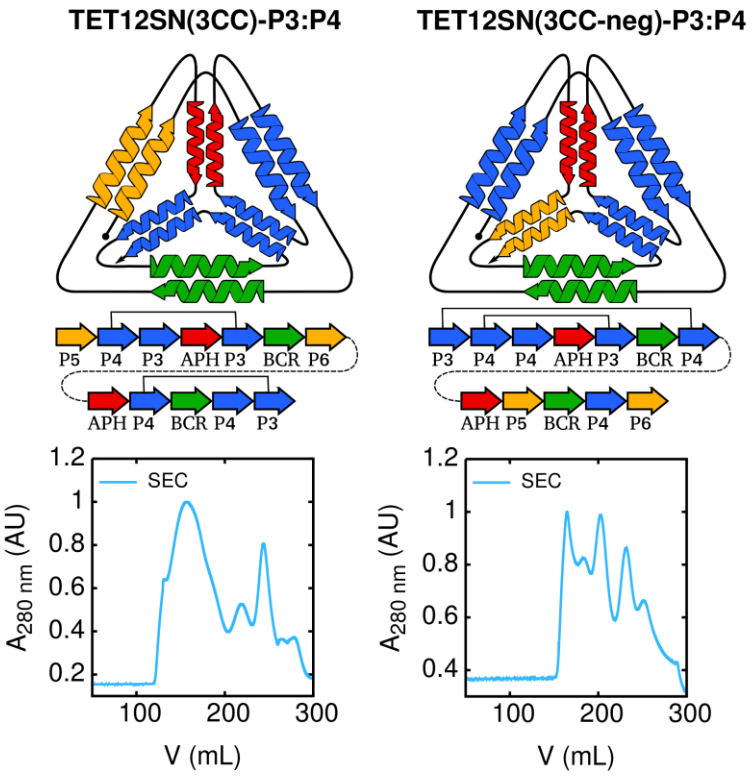

Supplementary Figure 18. Scheme and size-exclusion chromatograph after a Ni-NTA step for TET12SN(3CC)-P3:P4 (left) and TET12SN(3CC-neg)-P3:P4 (right). Source data are provided as a Source Data file.

Supplementary Tables

Peptide name	Amino acid sequence
APHSN	LEE ELKQLEE ELQAIEE QLACLQW KAQARKE KLAQLKE KL
BCRSN	DIEQ ELERAKQ SIRCLEQ EVNQERS RMQYLQT LLSK
GCNSN	QLED KVEELLS KNYHLEN EVERLKK LVGC
P3SN	EIQQLEE EISQLEQ KNSELKE KNQELKY GC
P4SN	KISQLKE KIQQLKQ ENQQLEE ENSQLEY GC
P5SN	ENSQLEE KISQLKQ KNSELKE EIQQLEY GC
P6SN	KNSELKE EICQLEE ENQQLEC KISELKY G
P7SN	EIQQLEE KNSQLKQ EISQLEE KNQELKY GC
P8SN	KISELKE ENQQLEQ KIQQLKE ENSQLEY GGC

Supplementary Table 1. Amino acid sequences of CC-forming peptides.

Circular permutation	segments	min	max	TCO	stdTCO	Probability of successful folding
**1.1**	ABCADECFEbDf	3	7	4.33	1.60	1.00
**1.2**	BCADECFEbDfA	3	9	5.33	2.21	1.00
**1.3**	CADECFEbDfAB	3	9	4.67	2.21	1.00
**1.4**	ADECFEbDfABC	3	9	5.33	2.49	1.00
**1.5**	DECFEbDfABCA	3	8	4.33	1.97	1.00
**1.6**	ECFEbDfABCAD	3	8	4.33	1.97	1.00
**1.7**	CFEbDfABCADE	3	9	5.33	2.49	1.00
**1.8**	FEbDfABCADEC	3	9	4.67	2.21	1.00
**1.9**	EbDfABCADECF	3	9	5.33	2.21	1.00
**1.10**	bDfABCADECFE	3	7	4.33	1.60	1.00
**1.11**	DfABCADECFEb	3	7	5.00	1.73	1.00
**1.12**	fABCADECFEbD	3	7	5.00	1.73	1.00

Supplementary Table 2. Probabilities of folding (P_F) as predicted by the deterministic folding model for the design case where two parallel edges in a CCPO tetrahedron are represented by the same coiled-coil building block. For each circular permutation deriving from topology 1 we report the segment arrangement, minimal (min), maximal (max) and average intra-chain distance (TCO) in the unfolded polypeptide chain along with the highest P_F observed during the sampling of all possible building block arrangements. In the segments column, same letters denote matching peptide pairs. Same-cased letters signify parallel CC pairs, while different-case letters mark antiparallel CCs.

Arrangement of peptides in the polypeptide sequence	P_F
APHsh-P5-BCR-P3-APHsh-P7-P4-P6-P5-P8-BCR-P6	1.00
APHsh-P7-BCR-P3-APHsh-P5-P4-P8-P5-P6-BCR-P6	1.00
APHsh-P7-BCR-P5-APHsh-P5-P6-P8-P3-P6-BCR-P4	1.00
APHsh-P3-BCR-P5-APHsh-P7-P6-P4-P6-P8-BCR-P5	1.00
APHsh-P5-BCR-P3-APHsh-P7-P4-P6-P6-P8-BCR-P5	0.97
APHsh-P5-BCR-P5-APHsh-P7-P6-P6-P3-P8-BCR-P4	0.90
APHsh-P7-BCR-P3-APHsh-P5-P4-P8-P6-P6-BCR-P5	0.00
APHsh-P7-BCR-P6-APHsh-P5-P5-P8-P3-P6-BCR-P4	0.00
APHsh-P3-BCR-P5-APHsh-P7-P6-P4-P5-P8-BCR-P6	0.00
APHsh-P5-BCR-P6-APHsh-P7-P5-P6-P3-P8-BCR-P4	0.00
APHsh-P6-BCR-P7-APHsh-P5-P8-P5-P3-P6-BCR-P4	0.00
APHsh-P5-BCR-P7-APHsh-P5-P8-P6-P3-P6-BCR-P4	0.00

Supplementary Table 3. Table of sampled building block arrangements containing two instances of the CC pair P5SN:P6SN and corresponding probabilities of folding (P_F) for the circular permutation 1.10.

Circular permutation	segments	min	max	TCO	stdTCO	Probability of successful folding
**1.1**	ABCADECFEbDf	3	7	4.33	1.60	1.00
**1.2**	BCADECFEbDfA	3	9	5.33	2.21	0.89
**1.3**	CADECFEbDfAB	3	9	4.67	2.21	0.80
**1.4**	ADECFEbDfABC	3	9	5.33	2.49	0.75
**1.5**	DECFEbDfABCA	3	8	4.33	1.97	1.00
**1.6**	ECFEbDfABCAD	3	8	4.33	1.97	1.00
**1.7**	CFEbDfABCADE	3	9	5.33	2.49	0.75
**1.8**	FEbDfABCADEC	3	9	4.67	2.21	0.80
**1.9**	EbDfABCADECF	3	9	5.33	2.21	0.89
**1.10**	bDfABCADECFE	3	7	4.33	1.60	1.00
**1.11**	DfABCADECFEb	3	7	5.00	1.73	1.00
**1.12**	fABCADECFEbD	3	7	5.00	1.73	1.00

Supplementary Table 4. Probabilities of folding (P_F) as predicted by the deterministic folding model for the design case where two pairs of CCPO tetrahedron edges are represented by the same coiled-coil building blocks. For each circular permutation deriving from topology 1 we report the highest P_F observed during the sampling of all possible building block arrangements.

Arrangement of peptides in the polypeptide sequence	P_F
APHsh-P5-BCR-P3-APHsh-P3-P4-P6-P5-P4-BCR-P6	1.00
APHsh-P3-BCR-P5-APHsh-P5-P6-P4-P3-P6-BCR-P4	1.00
APHsh-P6-BCR-P3-APHsh-P3-P4-P5-P5-P4-BCR-P6	0.97
APHsh-P4-BCR-P5-APHsh-P5-P6-P3-P3-P6-BCR-P4	0.97
APHsh-P3-BCR-P3-APHsh-P5-P4-P4-P5-P6-BCR-P6	0.90
APHsh-P5-BCR-P5-APHsh-P3-P6-P6-P3-P4-BCR-P4	0.90
APHsh-P3-BCR-P5-APHsh-P3-P6-P4-P5-P4-BCR-P6	0.00
APHsh-P3-BCR-P5-APHsh-P4-P6-P4-P5-P3-BCR-P6	0.00
APHsh-P5-BCR-P3-APHsh-P5-P4-P6-P3-P6-BCR-P4	0.00
APHsh-P5-BCR-P3-APHsh-P6-P4-P6-P3-P5-BCR-P4	0.00
APHsh-P3-BCR-P3-APHsh-P6-P4-P4-P5-P5-BCR-P6	0.00
APHsh-P3-BCR-P4-APHsh-P6-P3-P4-P5-P5-BCR-P6	0.00
APHsh-P5-BCR-P5-APHsh-P4-P6-P6-P3-P3-BCR-P4	0.00
APHsh-P5-BCR-P6-APHsh-P4-P5-P6-P3-P3-BCR-P4	0.00
APHsh-P6-BCR-P4-APHsh-P3-P3-P5-P5-P4-BCR-P6	0.00
APHsh-P4-BCR-P6-APHsh-P5-P5-P3-P3-P6-BCR-P4	0.00
APHsh-P5-BCR-P4-APHsh-P3-P3-P6-P5-P4-BCR-P6	0.00
APHsh-P3-BCR-P6-APHsh-P5-P5-P4-P3-P6-BCR-P4	0.00
APHsh-P3-BCR-P6-APHsh-P4-P5-P4-P5-P3-BCR-P6	0.00
APHsh-P5-BCR-P4-APHsh-P6-P3-P6-P3-P5-BCR-P4	0.00
APHsh-P3-BCR-P4-APHsh-P5-P3-P4-P5-P6-BCR-P6	0.00
APHsh-P5-BCR-P6-APHsh-P3-P5-P6-P3-P4-BCR-P4	0.00
APHsh-P3-BCR-P6-APHsh-P3-P5-P4-P5-P4-BCR-P6	0.00
APHsh-P5-BCR-P4-APHsh-P5-P3-P6-P3-P6-BCR-P4	0.00

Supplementary Table 5. Table of sampled building block arrangements containing two instances of CC pairs P5SN:P6SN and P3SN:P4SN with corresponding probabilities of folding (P_F) for the circular permutation 1.10.

Circular permutation	segments	min	max	TCO	stdTCO	Probability of successful folding
**1.1**	ABCADECFEbDf	3	7	4.33	1.60	1.00
**1.2**	BCADECFEbDfA	3	9	5.33	2.21	0.89
**1.3**	CADECFEbDfAB	3	9	4.67	2.21	0.77
**1.4**	ADECFEbDfABC	3	9	5.33	2.49	0.75
**1.5**	DECFEbDfABCA	3	8	4.33	1.97	1.00
**1.6**	ECFEbDfABCAD	3	8	4.33	1.97	1.00
**1.7**	CFEbDfABCADE	3	9	5.33	2.49	0.75
**1.8**	FEbDfABCADEC	3	9	4.67	2.21	0.77
**1.9**	EbDfABCADECF	3	9	5.33	2.21	0.89
**1.10**	bDfABCADECFE	3	7	4.33	1.60	1.00
**1.11**	DfABCADECFEb	3	7	5.00	1.73	0.93
**1.12**	fABCADECFEbD	3	7	5.00	1.73	0.93

Supplementary Table 6. Probabilities of folding (P_F) as predicted by the deterministic folding model for the design case where three CC modules occur twice in the CCPO tetrahedron. For each circular permutation deriving from topology 1 we report the highest P_F observed during the sampling of all possible building block arrangements.

Arrangement of peptides in the polypeptide sequence	P_F
APHsh-P5-APHsh-P3-APHsh-P3-P4-P6-P5-P4-APHsh-P6	1.00
APHsh-P3-APHsh-P5-APHsh-P5-P6-P4-P3-P6-APHsh-P4	1.00
APHsh-P4-APHsh-P5-APHsh-P5-P6-P3-P3-P6-APHsh-P4	0.97
APHsh-P6-APHsh-P3-APHsh-P3-P4-P5-P5-P4-APHsh-P6	0.97
APHsh-P3-APHsh-P3-APHsh-P5-P4-P4-P5-P6-APHsh-P6	0.88
APHsh-P5-APHsh-P5-APHsh-P3-P6-P6-P3-P4-APHsh-P4	0.88
APHsh-P5-APHsh-P3-APHsh-P5-P4-P6-P3-P6-APHsh-P4	0.00
APHsh-P6-APHsh-P3-APHsh-P5-P4-P5-P3-P6-APHsh-P4	0.00
APHsh-P3-APHsh-P5-APHsh-P3-P6-P4-P5-P4-APHsh-P6	0.00
APHsh-P4-APHsh-P5-APHsh-P3-P6-P3-P5-P4-APHsh-P6	0.00
APHsh-P3-APHsh-P3-APHsh-P5-P4-P4-P6-P6-APHsh-P5	0.00
APHsh-P4-APHsh-P3-APHsh-P5-P4-P3-P6-P6-APHsh-P5	0.00
APHsh-P5-APHsh-P5-APHsh-P3-P6-P6-P4-P4-APHsh-P3	0.00
APHsh-P6-APHsh-P5-APHsh-P3-P6-P5-P4-P4-APHsh-P3	0.00
APHsh-P4-APHsh-P6-APHsh-P5-P5-P3-P3-P6-APHsh-P4	0.00
APHsh-P6-APHsh-P4-APHsh-P3-P3-P5-P5-P4-APHsh-P6	0.00
APHsh-P3-APHsh-P6-APHsh-P5-P5-P4-P3-P6-APHsh-P4	0.00
APHsh-P5-APHsh-P4-APHsh-P3-P3-P6-P5-P4-APHsh-P6	0.00
APHsh-P6-APHsh-P3-APHsh-P5-P4-P5-P4-P6-APHsh-P3	0.00
APHsh-P4-APHsh-P5-APHsh-P3-P6-P3-P6-P4-APHsh-P5	0.00
APHsh-P4-APHsh-P3-APHsh-P5-P4-P3-P5-P6-APHsh-P6	0.00
APHsh-P6-APHsh-P5-APHsh-P3-P6-P5-P3-P4-APHsh-P4	0.00
APHsh-P5-APHsh-P3-APHsh-P5-P4-P6-P4-P6-APHsh-P3	0.00
APHsh-P3-APHsh-P5-APHsh-P3-P6-P4-P6-P4-APHsh-P5	0.00

Supplementary Table 7. Table of sampled building block arrangements containing two instances of CC pairs P5SN:P6SN, P3SN:P4SN and APHSN homodimer with corresponding probabilities of folding (P_F) for the circular permutation 1.10.

Circular permutation	segments	min	max	TCO	stdTCO	Probability of successful folding
**1.1**	ABCADECFEbDf	3	7	4.33	1.60	0.83
**1.2**	BCADECFEbDfA	3	9	5.33	2.21	0.53
**1.3**	CADECFEbDfAB	3	9	4.67	2.21	0.40
**1.4**	ADECFEbDfABC	3	9	5.33	2.49	0.67
**1.5**	DECFEbDfABCA	3	8	4.33	1.97	0.92
**1.6**	ECFEbDfABCAD	3	8	4.33	1.97	0.92
**1.7**	CFEbDfABCADE	3	9	5.33	2.49	0.67
**1.8**	FEbDfABCADEC	3	9	4.67	2.21	0.40
**1.9**	EbDfABCADECF	3	9	5.33	2.21	0.53
**1.10**	bDfABCADECFE	3	7	4.33	1.60	0.83
**1.11**	DfABCADECFEb	3	7	5.00	1.73	0.87
**1.12**	fABCADECFEbD	3	7	5.00	1.73	0.87

Supplementary Table 8. Probabilities of folding (P_F) as predicted by the deterministic folding model for the design case where three parallel edges in a CCPO tetrahedron are represented by the same coiled-coil building block. For each circular permutation deriving from topology 1 we report the highest P_F observed during the sampling of all possible building block arrangements.

Arrangement of peptides in the polypeptide sequence	P_F
P3-P6-P5-APHsh-P5-BCR-P4-APHsh-P6-BCR-P6-P5	0.93
P5-P3-P5-APHsh-P4-BCR-P6-APHsh-P6-BCR-P6-P5	0.69
P5-P5-P3-APHsh-P6-BCR-P6-APHsh-P6-BCR-P4-P5	0.63
P5-P3-P6-APHsh-P4-BCR-P6-APHsh-P5-BCR-P5-P6	0.00
P3-P5-P5-APHsh-P6-BCR-P4-APHsh-P6-BCR-P6-P5	0.00
P5-P6-P3-APHsh-P5-BCR-P6-APHsh-P6-BCR-P4-P5	0.00
P5-P6-P5-APHsh-P5-BCR-P6-APHsh-P3-BCR-P6-P4	0.00
P5-P3-P6-APHsh-P4-BCR-P6-APHsh-P6-BCR-P5-P5	0.00
P3-P5-P5-APHsh-P6-BCR-P4-APHsh-P5-BCR-P6-P6	0.00
P5-P6-P3-APHsh-P5-BCR-P6-APHsh-P5-BCR-P4-P6	0.00
P5-P5-P5-APHsh-P6-BCR-P6-APHsh-P3-BCR-P6-P4	0.00
P5-P3-P5-APHsh-P4-BCR-P6-APHsh-P5-BCR-P6-P6	0.00
P5-P5-P3-APHsh-P6-BCR-P6-APHsh-P5-BCR-P4-P6	0.00
P3-P6-P5-APHsh-P5-BCR-P4-APHsh-P5-BCR-P6-P6	0.00
P5-P5-P6-APHsh-P6-BCR-P6-APHsh-P3-BCR-P5-P4	0.00
P5-P6-P6-APHsh-P5-BCR-P6-APHsh-P3-BCR-P5-P4	0.00

Supplementary Table 9. Table of sampled building block arrangements containing three instances of the CC pair P5SN:P6SN with corresponding probabilities of folding (P_F) for the circular permutation 1.5.

Protein name	Amino acid sequence
TET12SN(2CC)	MLEEELKQLEEELQAIEEQLAQLQWKAQARKEKLAQLKEKLGSGPGSPED<br>ENSQLEEKISQLKQKNSELKEEIQQLEYGSGPGDIEQELERAKESIRRLE<br>QEVNQERSRMQYLQTLLEKGSGPGSPEDEIQQLEEEISQLEQKNSELKEK<br>NQELKYGSGPGLEEELKQLEEELQAIEEQLAQLQWKAQARKEKLAQLKEK<br>LGSGPGSPEDEIQQLEEKNSQLKQEISQLEEKNQELKYGSGPGSPEDKIS<br>QLKEKIQQLKQENQQLEEENSQLEYGSGPGSPEDKNSELKEEIQQLEEEN<br>QQLEEKISELKYGSGPGSPEDENSQLEEKISQLKQKNSELKEEIQQLEYG<br>SGPGSPEDKISELKEENQQLEQKIQQLKEENSQLEYGSGPGDIEQELERA<br>KESIRRLEQEVNQERSRMQYLQTLLEKGSGPGSPEDKNSELKEEIQQLEE<br>ENQQLEEKISELKYGLEHHHHHHHH
TET12SN(22CC)	MLEEELKQLEEELQAIEEQLAQLQWKAQARKEKLAQLKEKLGSGPGSPED<br>ENSQLEEKISQLKQKNSELKEEIQQLEYGSGPGDIEQELERAKESIRRLE<br>QEVNQERSRMQYLQTLLEKGSGPGSPEDEIQQLEEEISQLEQKNSELKEK<br>NQELKYGSGPGLEEELKQLEEELQAIEEQLAQLQWKAQARKEKLAQLKEK<br>LGSGPGSPEDEIQQLEEEISQLEQKNSELKEKNQELKYGSGPGSPEDKIS<br>QLKEKIQQLKQENQQLEEENSQLEYGSGPGSPEDKNSELKEEIQQLEEEN<br>QQLEEKISELKYGSGPGSPEDENSQLEEKISQLKQKNSELKEEIQQLEYG<br>SGPGSPEDKISQLKEKIQQLKQENQQLEEENSQLEYGSGPGDIEQELERA<br>KESIRRLEQEVNQERSRMQYLQTLLEKGSGPGSPEDKNSELKEEIQQLEE<br>ENQQLEEKISELKYGLEHHHHHHHH
TET12SN(222CC)	MLEEELKQLEEELQAIEEQLAQLQWKAQARKEKLAQLKEKLGSGPGSPED<br>ENSQLEEKISQLKQKNSELKEEIQQLEYGSGPGLEEELKQLEEELQAIEE<br>QLAQLQWKAQARKEKLAQLKEKLGSGPGSPEDEIQQLEEEISQLEQKNSE<br>LKEKNQELKYGSGPGLEEELKQLEEELQAIEEQLAQLQWKAQARKEKLAQ<br>LKEKLGSGPGSPEDEIQQLEEEISQLEQKNSELKEKNQELKYGSGPGSPE<br>DKISQLKEKIQQLKQENQQLEEENSQLEYGSGPGSPEDKNSELKEEIQQL<br>EEENQQLEEKISELKYGSGPGSPEDENSQLEEKISQLKQKNSELKEEIQQ<br>LEYGSGPGSPEDKISQLKEKIQQLKQENQQLEEENSQLEYGSGPGLEEEL<br>KQLEEELQAIEEQLAQLQWKAQARKEKLAQLKEKLGSGPGSPEDKNSELK<br>EEIQQLEEENQQLEEKISELKYGLEHHHHHHHH
TET12SN(3CC)	MSPEDEIQQLEEEISQLEQKNSELKEKNQELKYGSGPGSPEDKNSELKEE<br>IQQLEEENQQLEEKISELKYGSGPGSPEDENSQLEEKISQLKQKNSELKE<br>EIQQLEYGSGPGLEEELKQLEEELQAIEEQLAQLQWKAQARKEKLAQLKE<br>KLGSGPGSPEDENSQLEEKISQLKQKNSELKEEIQQLEYGSGPGDIEQEL<br>ERAKESIRRLEQEVNQERSRMQYLQTLLEKGSGPGSPEDKISQLKEKIQQ<br>LKQENQQLEEENSQLEYGSGPGLEEELKQLEEELQAIEEQLAQLQWKAQA<br>RKEKLAQLKEKLGSGPGSPEDKNSELKEEIQQLEEENQQLEEKISELKYG<br>SGPGDIEQELERAKESIRRLEQEVNQERSRMQYLQTLLEKGSGPGSPEDK<br>NSELKEEIQQLEEENQQLEEKISELKYGSGPGSPEDENSQLEEKISQLKQ<br>KNSELKEEIQQLEYGLEHHHHHHHH
TET12SN(3CC)-neg	MSPEDENSQLEEKISQLKQKNSELKEEIQQLEYGSGPGSPEDKNSELKEE<br>IQQLEEENQQLEEKISELKYGSGPGSPEDKNSELKEEIQQLEEENQQLEE<br>KISELKYGSGPGLEEELKQLEEELQAIEEQLAQLQWKAQARKEKLAQLKE<br>KLGSGPGSPEDENSQLEEKISQLKQKNSELKEEIQQLEYGSGPGDIEQEL<br>ERAKESIRRLEQEVNQERSRMQYLQTLLEKGSGPGSPEDKNSELKEEIQQ<br>LEEENQQLEEKISELKYGSGPGLEEELKQLEEELQAIEEQLAQLQWKAQA<br>RKEKLAQLKEKLGSGPGSPEDEIQQLEEEISQLEQKNSELKEKNQELKYG<br>SGPGDIEQELERAKESIRRLEQEVNQERSRMQYLQTLLEKGSGPGSPEDE<br>NSQLEEKISQLKQKNSELKEEIQQLEYGSGPGSPEDKISQLKEKIQQLKQ<br>ENQQLEEENSQLEYGLEHHHHHHHH

TET12SN(3CC)-P3:P4	MSPEDENSQLEEKISQLKQKNSELKEEIQQLEYGSGPGSPEDKISQLKEK<br>IQQLKQENQQLEEENSQLEYGSGPGSPEDEIQQLEEEISQLEQKNSELKE<br>KNQELKYGSGPGLEEELKQLEEELQAIEEQLAQLQWKAQARKEKLAQLKE<br>KLGSGPGSPEDEIQQLEEEISQLEQKNSELKEKNQELKYGSGPGDIEQEL<br>ERAKESIRRLEQEVNQERSRMQYLQTLLEKGSGPGSPEDKNSELKEEIQQ<br>LEEENQQLEEKISELKYGSGPGLEEELKQLEEELQAIEEQLAQLQWKAQA<br>RKEKLAQLKEKLGSGPGSPEDKISQLKEKIQQLKQENQQLEEENSQLEYG<br>SGPGDIEQELERAKESIRRLEQEVNQERSRMQYLQTLLEKGSGPGSPEDK<br>ISQLKEKIQQLKQENQQLEEENSQLEYGSGPGSPEDEIQQLEEEISQLEQ<br>KNSELKEKNQELKYGLEHHHHHHHH
TET12SN(3CC-neg)-P3:P4	MSPEDEIQQLEEEISQLEQKNSELKEKNQELKYGSGPGSPEDKISQLKEK<br>IQQLKQENQQLEEENSQLEYGSGPGSPEDKISQLKEKIQQLKQENQQLEE<br>ENSQLEYGSGPGLEEELKQLEEELQAIEEQLAQLQWKAQARKEKLAQLKE<br>KLGSGPGSPEDEIQQLEEEISQLEQKNSELKEKNQELKYGSGPGDIEQEL<br>ERAKESIRRLEQEVNQERSRMQYLQTLLEKGSGPGSPEDKISQLKEKIQQ<br>LKQENQQLEEENSQLEYGSGPGLEEELKQLEEELQAIEEQLAQLQWKAQA<br>RKEKLAQLKEKLGSGPGSPEDENSQLEEKISQLKQKNSELKEEIQQLEYG<br>SGPGDIEQELERAKESIRRLEQEVNQERSRMQYLQTLLEKGSGPGSPEDE<br>IQQLEEEISQLEQKNSELKEKNQELKYGSGPGSPEDKNSELKEEIQQLEE<br>ENQQLEEKISELKYGLEHHHHHHHH
TET12SN-GCNcys	MLEEELKQLEEELQAIEEQLAQLQWKAQARKEKLAQLKEKLSGPGSPEDE<br>IQQLEEEISQLEQKNSELKEKNQELKYGSGPGDIEQELERAKESIRRLEQ<br>EVNQERSRMQYLQTLLEKSGPGQLEDKVEELLSKNYHLENEVERLKKLVG<br>CGPGLEEELKQLEEELQAIEEQLAQLQWKAQARKEKLAQLKEKLSGPGSP<br>EDEIQQLEEKNSQLKQEISQLEEKNQELKYGSGPGQLEDKVEELLSKNYH<br>LENEVERLKKLVGCGPGSPEDKISQLKEKIQQLKQENQQLEEENSQLEYG<br>SGPGSPEDENSQLEEKISQLKQKNSELKEEIQQLEYGSGPGSPEDKISEL<br>KEENQQLEQKIQQLKEENSQLEYGSGPGDIEQELERAKESIRRLEQEVNQ<br>ERSRMQYLQTLLEKSGPGSPEDKNSELKEEIQQLEEENQQLEEKISELKY<br>GLEHHHHHHHH
TET12SN-P7P8cys	MLEEELKQLEEELQAIEEQLAQLQWKAQARKEKLAQLKEKLSGPGSPEDE<br>IQQLEEEISQLEQKNSELKEKNQELKYGSGPGDIEQELERAKESIRRLEQ<br>EVNQERSRMQYLQTLLEKSGPGQLEDKVEELLSKNYHLENEVERLKKLVG<br>SGPGLEEELKQLEEELQAIEEQLAQLQWKAQARKEKLAQLKEKLSGPGSP<br>EDEIQQLEEKNSQLKQEISQLEEKNQELKYGCGPGQLEDKVEELLSKNYH<br>LENEVERLKKLVGSGPGSPEDKISQLKEKIQQLKQENQQLEEENSQLEYG<br>SGPGSPEDENSQLEEKISQLKQKNSELKEEIQQLEYGSGPGSPEDKISEL<br>KEENQQLEQKIQQLKEENSQLEYGCGPGDIEQELERAKESIRRLEQEVNQ<br>ERSRMQYLQTLLEKSGPGSPEDKNSELKEEIQQLEEENQQLEEKISELKY<br>GLEHHHHHHHH
TET12SN-P5P6cys	MLEEELKQLEEELQAIEEQLAQLQWKAQARKEKLAQLKEKLSGPGSPEDE<br>IQQLEEEISQLEQKNSELKEKNQELKYGSGPGDIEQELERAKESIRRLEQ<br>EVNQERSRMQYLQTLLEKSGPGQLEDKVEELLSKNYHLENEVERLKKLVG<br>SGPGLEEELKQLEEELQAIEEQLAQLQWKAQARKEKLAQLKEKLSGPGSP<br>EDEIQQLEEKNSQLKQEISQLEEKNQELKYGSGPGQLEDKVEELLSKNYH<br>LENEVERLKKLVGSGPGSPEDKISQLKEKIQQLKQENQQLEEENSQLEYG<br>SGPGSPEDENSQLEEKISQLKQKNSELKEEIQQLEYGCGPGSPEDKISEL<br>KEENQQLEQKIQQLKEENSQLEYGSGPGDIEQELERAKESIRRLEQEVNQ<br>ERSRMQYLQTLLEKSGPGSPEDKNSELKEEIQQLEEENQQLEEKISELKY<br>GCLEHHHHHHHH

TET12SN-BCRcys	MLEEELKQLEEELQAIEEQLAQLQWKAQARKEKLAQLKEKLSGPGSPEDE<br>IQQLEEEISQLEQKNSELKEKNQELKYGSGPGDIEQELERAKESIRCLEQ<br>EVNQERSRMQYLQTLLEKSGPGQLEDKVEELLSKNYHLENEVERLKKLVG<br>SGPGLEEELKQLEEELQAIEEQLAQLQWKAQARKEKLAQLKEKLSGPGSP<br>EDEIQQLEEKNSQLKQEISQLEEKNQELKYGSGPGQLEDKVEELLSKNYH<br>LENEVERLKKLVGSGPGSPEDKISQLKEKIQQLKQENQQLEEENSQLEYG<br>SGPGSPEDENSQLEEKISQLKQKNSELKEEIQQLEYGSGPGSPEDKISEL<br>KEENQQLEQKIQQLKEENSQLEYGSGPGDIEQELERAKESIRCLEQEVNQ<br>ERSRMQYLQTLLEKSGPGSPEDKNSELKEEIQQLEEENQQLEEKISELKY<br>GLEHHHHHHHH
TET12SN-P3P4cys	MLEEELKQLEEELQAIEEQLAQLQWKAQARKEKLAQLKEKLSGPGSPEDE<br>IQQLEEEISQLEQKNSELKEKNQELKYGCGPGDIEQELERAKESIRRLEQ<br>EVNQERSRMQYLQTLLEKSGPGQLEDKVEELLSKNYHLENEVERLKKLVG<br>SGPGLEEELKQLEEELQAIEEQLAQLQWKAQARKEKLAQLKEKLSGPGSP<br>EDEIQQLEEKNSQLKQEISQLEEKNQELKYGSGPGQLEDKVEELLSKNYH<br>LENEVERLKKLVGSGPGSPEDKISQLKEKIQQLKQENQQLEEENSQLEYG<br>CGPGSPEDENSQLEEKISQLKQKNSELKEEIQQLEYGSGPGSPEDKISEL<br>KEENQQLEQKIQQLKEENSQLEYGSGPGDIEQELERAKESIRRLEQEVNQ<br>ERSRMQYLQTLLEKSGPGSPEDKNSELKEEIQQLEEENQQLEEKISELKY<br>GLEHHHHHHHH
TET12SN-APHcys	MLEEELKQLEEELQAIEEQLACLQWKAQARKEKLAQLKEKLSGPGSPEDE<br>IQQLEEEISQLEQKNSELKEKNQELKYGSGPGDIEQELERAKESIRRLEQ<br>EVNQERSRMQYLQTLLEKSGPGQLEDKVEELLSKNYHLENEVERLKKLVG<br>SGPGLEEELKQLEEELQAIEEQLACLQWKAQARKEKLAQLKEKLSGPGSP<br>EDEIQQLEEKNSQLKQEISQLEEKNQELKYGSGPGQLEDKVEELLSKNYH<br>LENEVERLKKLVGSGPGSPEDKISQLKEKIQQLKQENQQLEEENSQLEYG<br>SGPGSPEDENSQLEEKISQLKQKNSELKEEIQQLEYGSGPGSPEDKISEL<br>KEENQQLEQKIQQLKEENSQLEYGSGPGDIEQELERAKESIRRLEQEVNQ<br>ERSRMQYLQTLLEKSGPGSPEDKNSELKEEIQQLEEENQQLEEKISELKY<br>GLEHHHHHHHH

Supplementary Table 10. Protein sequences of characterised CCPO tetrahedra.

Construct name	DNA sequence
TET12SN	CATATGCTTGAGGAAGAACTGAAGCAGTTGGAAGAAGAGTTGCAAGCGATCGAAGAGCAGTTGGCGCAGCTGCAGTGGAAGGCACAGGCGCGCAAAGAGAAGTTAG<br>CGCAGTTAAAAGAAAAGTTGGGCTCTGGGCCTGGCTCACCGGAAGACGAGATCCAGCAGTTGGAAGAAGAAATCTCTCAGCTGGAGCAGAAAAACAGCGAACTGAA<br>AGAAAAAAACCAGGAACTGAAATATGGTAGCGGCCCGGGTGATATTGAGCAGGAACTGGAACGTGCCAAAGAATCGATTCGTCGGTTAGAACAGGAGGTTAACCAA<br>GAACGCTCGCGCATGCAATACTTGCAAACTTTGCTTGAAAAAGGGTCCGGTCCTGGACAACTGGAGGACAAGGTGGAAGAATTGCTCTCCAAAAACTATCACTTGG<br>AAAACGAAGTTGAGCGCCTGAAGAAACTGGTAGGTTCAGGCCCGGGGCTGGAGGAGGAACTGAAGCAGCTGGAAGAAGAACTGCAGGCGATTGAAGAGCAACTGGC<br>ACAGTTACAGTGGAAGGCCCAGGCGCGTAAAGAGAAATTAGCGCAGTTGAAAGAGAAGCTGGGATCAGGGCCAGGAAGCCCGGAAGATGAAATCCAGCAACTTGAA<br>GAGAAAAATTCACAACTCAAGCAGGAAATTTCCCAGCTTGAAGAAAAAAATCAGGAATTAAAATATGGTTCTGGCCCCGGTCAACTGGAAGACAAGGTCGAAGAGC<br>TTTTGTCCAAAAACTACCATTTAGAAAATGAAGTGGAACGCCTTAAAAAACTGGTAGGTAGCGGACCGGGTTCTCCTGAGGATAAAATCTCGCAGTTGAAAGAAAA<br>AATTCAGCAACTGAAACAAGAGAATCAACAGCTGGAAGAAGAGAATTCGCAGCTGGAATACGGCTCAGGTCCTGGTTCTCCGGAAGATGAAAACAGCCAGTTAGAA<br>GAAAAAATTAGTCAGCTGAAGCAGAAAAACTCGGAGCTGAAAGAAGAGATTCAGCAGCTGGAATACGGTTCCGGCCCGGGGTCCCCTGAAGATAAAATTTCCGAGC<br>TGAAAGAAGAAAATCAGCAGCTGGAACAGAAGATTCAACAACTGAAAGAAGAAAACTCGCAACTGGAATATGGTTCGGGACCGGGGGACATTGAGCAAGAGCTGGA<br>ACGCGCCAAAGAATCCATTCGTCGTTTGGAACAAGAAGTTAATCAGGAACGCTCACGTATGCAATATCTGCAGACCCTGCTGGAGAAAGGTTCCGGTCCAGGTAGC<br>CCCGAGGATAAAAATTCAGAACTGAAAGAAGAAATTCAGCAACTGGAAGAAGAAAATCAACAATTAGAAGAGAAAATCTCGGAGCTGAAGTATGGCCTCGAGCATC<br>ATCATCACCATCATCATCATTAA
TET12SN(2CC)	CATATGCTTGAGGAAGAACTGAAGCAGTTGGAAGAAGAGTTGCAAGCGATCGAAGAGCAGTTGGCGCAGCTGCAGTGGAAGGCACAGGCGCGCAAAGAGAAGTTAG<br>CGCAGTTAAAAGAAAAGTTGGGCTCTGGGCCTGGCTCTCCGGAAGATGAAAACAGCCAGTTAGAAGAAAAAATTAGTCAGCTGAAGCAGAAAAACTCGGAGCTGAA<br>AGAAGAGATTCAGCAGCTGGAATACGGTAGCGGCCCGGGTGATATTGAGCAGGAACTGGAACGTGCCAAAGAATCGATTCGTCGGTTAGAACAGGAGGTTAACCAA<br>GAACGCTCGCGCATGCAATACTTGCAAACTTTGCTTGAAAAAGGGTCCGGTCCTGGATCACCGGAAGACGAGATCCAGCAGTTGGAAGAAGAAATCTCTCAGCTGG<br>AGCAGAAAAACAGCGAACTGAAAGAAAAAAACCAGGAACTGAAATATGGTTCAGGCCCGGGGCTGGAGGAGGAACTGAAGCAGCTGGAAGAAGAACTGCAGGCGAT<br>TGAAGAGCAACTGGCACAGTTACAGTGGAAGGCCCAGGCGCGTAAAGAGAAATTAGCGCAGTTGAAAGAGAAGCTGGGATCAGGGCCAGGAAGCCCGGAAGATGAA<br>ATCCAGCAACTTGAAGAGAAAAATTCACAACTCAAGCAGGAAATTTCCCAGCTTGAAGAAAAAAATCAGGAATTAAAATATGGTTCTGGCCCCGGTTCTCCTGAGG<br>ATAAAATCTCGCAGTTGAAAGAAAAAATTCAGCAACTGAAACAAGAGAATCAACAGCTGGAAGAAGAGAATTCGCAGCTGGAATACGGTAGCGGACCGGGTAGCCC<br>CGAGGATAAAAATTCAGAACTGAAAGAAGAAATTCAGCAACTGGAAGAAGAAAATCAACAATTAGAAGAGAAAATCTCGGAGCTGAAGTATGGCTCAGGTCCTGGT<br>TCTCCGGAAGATGAAAACAGCCAGTTAGAAGAAAAAATTAGTCAGCTGAAGCAGAAAAACTCGGAGCTGAAAGAAGAGATTCAGCAGCTGGAATACGGTTCCGGCC<br>CGGGGTCCCCTGAAGATAAAATTTCCGAGCTGAAAGAAGAAAATCAGCAGCTGGAACAGAAGATTCAACAACTGAAAGAAGAAAACTCGCAACTGGAATATGGTTC<br>GGGACCGGGGGACATTGAGCAAGAGCTGGAACGCGCCAAAGAATCCATTCGTCGTTTGGAACAAGAAGTTAATCAGGAACGCTCACGTATGCAATATCTGCAGACC<br>CTGCTGGAGAAAGGTTCCGGTCCAGGTAGCCCCGAGGATAAAAATTCAGAACTGAAAGAAGAAATTCAGCAACTGGAAGAAGAAAATCAACAATTAGAAGAGAAAA<br>TCTCGGAGCTGAAGTATGGCCTCGAGCATCATCATCACCATCATCATCATTAA
TET12SN(22CC)	CATATGCTTGAGGAAGAGCTAAAACAATTGGAAGAGGAACTACAGGCAATTGAGGAGCAACTGGCACAACTGCAATGGAAAGCTCAAGCCCGAAAAGAGAAGCTGG<br>CCCAATTGAAGGAGAAGTTGTCAGGGCCAGGTTCTCCTGAGGACGAGAACTCTCAGCTTGAGGAAAAGATCAGTCAGTTGAAGCAAAAGAACTCTGAACTTAAAGA<br>GGAAATTCAGCAGCTTGAATACGGATCGGGTCCTGGGGATATAGAACAAGAGTTAGAGCGTGCTAAGGAAAGCATTCGTAGGTTAGAACAGGAAGTCAACCAGGAG<br>AGATCACGAATGCAATATCTACAGACCTTGCTTGAGAAATCAGGACCAGGATCGCCTGAAGATGAGATCCAGCAGCTAGAGGAAGAGATTTCCCAACTGGAGCAGA<br>AGAACTCTGAGCTGAAGGAGAAGAATCAAGAACTTAAATACGGATCCGGTCCGGGTCTCGAGGAAGAACTCAAACAGCTGGAAGAAGAATTGCAGGCCATTGAGGA<br>ACAACTTGCTCAACTGCAGTGGAAGGCTCAAGCTAGGAAAGAGAAACTCGCCCAATTAAAAGAGAAACTTTCTGGTCCTGGATCTCCTGAGGATGAGATTCAACAG<br>TTAGAGGAAGAGATATCGCAGTTAGAGCAGAAGAATAGTGAACTCAAGGAGAAGAATCAAGAATTGAAGTATGGATCGGGTCCTGGAAGTCCAGAGGATAAGATTT<br>CTCAGCTTAAAGAGAAAATACAACAGCTTAAGCAGGAGAACCAACAGCTCGAAGAGGAGAACTCTCAACTAGAGTACGGTTCAGGGCCTGGCTCTCCTGAGGACAA<br>GAATTCCGAGCTTAAAGAGGAGATCCAGCAGTTGGAAGAGGAGAACCAGCAGTTAGAGGAGAAAATCAGTGAATTGAAGTACGGATCTGGACCAGGGTCTCCAGAG<br>GACGAAAATTCCCAGCTTGAGGAGAAAATCAGTCAACTTAAACAGAAGAATTCAGAACTAAAAGAGGAAATACAACAGCTTGAGTACGGATCTGGTCCAGGAAGCC<br>CAGAAGATAAGATTTCTCAACTGAAAGAGAAAATACAACAGCTAAAACAAGAGAATCAGCAACTAGAGGAAGAAAATTCTCAGCTTGAATACGGGTCAGGCCCAGG<br>TGATATCGAGCAAGAGCTCGAGCGAGCCAAAGAGTCCATCAGGCGTTTAGAACAAGAGGTGAACCAGGAGCGTAGTCGAATGCAATACCTGCAAACTCTGTTGGAG<br>AAGTCGGGGCCTGGTTCGCCAGAAGACAAGAACAGTGAGTTGAAGGAAGAAATACAACAATTGGAAGAAGAAAATCAACAGTTAGAGGAAAAGATATCCGAACTCA<br>AATACGGTTTGGAACATCATCACCATCATCATCATCATTAA

TET12SN(222CC)	CACGCATATGCTTGAGGAAGAACTGAAGCAGTTGGAAGAAGAGTTGCAAGCGATCGAAGAGCAGTTGGCGCAGCTGCAGTGGAAGGCACAGGCGCGCAAAGAGAAG TTAGCGCAGTTAAAAGAAAAGTTGGGCTCAGGTCCGGGTTCGCCAGAGGATGAGAATAGCCAACTTGAAGAAAAGATCAGTCAATTGAAGCAAAAAAACAGTGAAC TGAAGGAGGAAATTCAACAGTTAGAGTACGGTTCAGGTCCGGGCTTAGAGGAGGAGTTGAAACAGTTGGAAGAAGAGCTTCAAGCAATCGAGGAGCAGTTAGCTCA ATTGCAGTGGAAAGCTCAAGCCCGCAAGGAAAAGCTGGCGCAGCTGAAAGAGAAGCTGGGGTCTGGCCCGGGCTCACCGGAAGACGAGATCCAGCAGTTGGAAGAA GAAATCTCTCAGCTGGAGCAGAAAAACAGCGAACTGAAAGAAAAAAACCAGGAACTGAAATATGGTAGCGGTCCTGGCCTGGAGGAGGAACTGAAGCAGCTGGAAG AAGAACTGCAGGCGATTGAAGAGCAACTGGCACAGTTACAGTGGAAGGCCCAGGCGCGTAAAGAGAAATTAGCGCAGTTGAAAGAGAAGCTGGGTTCGGGTCCGGG TTCACCCGAAGATGAAATTCAGCAGCTGGAGGAGGAGATTAGCCAGTTAGAACAAAAAAACTCCGAACTGAAAGAGAAAAATCAGGAACTTAAATATGGATCCGGC CCGGGTTCTCCTGAGGATAAAATCTCGCAGTTGAAAGAAAAAATTCAGCAACTGAAACAAGAGAATCAACAGCTGGAAGAAGAGAATTCGCAGCTGGAATACGGTA GCGGTCCGGGCTCTCCGGAAGACAAGAATTCTGAGCTGAAGGAAGAAATTCAACAATTAGAAGAGGAGAACCAACAACTGGAGGAGAAAATTTCAGAGCTGAAATA TGGCAGTGGCCCGGGTTCTCCGGAAGATGAAAACAGCCAGTTAGAAGAAAAAATTAGTCAGCTGAAGCAGAAAAACTCGGAGCTGAAAGAAGAGATTCAGCAGCTG GAATACGGTAGCGGCCCTGGTAGTCCTGAGGATAAGATTTCGCAGCTGAAGGAGAAGATTCAACAGTTGAAGCAAGAGAATCAACAATTAGAGGAGGAAAATTCAC AGTTGGAGTATGGTAGCGGACCGGGCCTGGAAGAGGAGCTGAAGCAATTGGAAGAAGAATTACAGGCGATTGAGGAGCAATTGGCACAGCTTCAGTGGAAAGCTCA AGCACGCAAGGAAAAATTAGCCCAACTTAAAGAGAAGTTAGGTAGCGGTCCTGGTAGCCCCGAGGATAAAAATTCAGAACTGAAAGAAGAAATTCAGCAACTGGAA GAAGAAAATCAACAATTAGAAGAGAAAATCTCGGAGCTGAAGTATCTCGAGCATCATCATCACCATCATCATCATTAA
TET12SN(3CC)	CATATGAGTCCTGAGGATGAGATCCAACAGTTAGAGGAAGAAATATCGCAGTTAGAGCAAAAGAATTCAGAACTGAAGGAAAAGAATCAAGAACTAAAGTATGGAA GTGGTCCCGGCTCACCAGAGGACAAGAATTCAGAGCTGAAGGAAGAAATTCAACAGCTTGAGGAAGAAAATCAGCAGCTTGAAGAAAAGATATCGGAATTAAAGTA TGGTTCGGGTCCTGGTAGCCCCGAAGACGAAAATAGTCAATTAGAGGAAAAGATCTCACAATTAAAACAGAAGAATAGTGAGTTAAAAGAGGAGATTCAGCAATTG GAGTACGGCTCCGGACCTGGCCTGGAAGAAGAACTAAAACAGCTAGAGGAAGAGTTACAAGCAATCGAAGAACAACTAGCACAATTACAATGGAAAGCTCAGGCCA GGAAGGAAAAGCTCGCACAACTAAAGGAAAAGCTTAGCGGCCAGGCTCGCCAGAAGATGAAAACTCCCAATTAGAAGAGAAGATCTCACAATTGAAGCAGAAGAA TTCCGAACTAAAGGAAGAGATCCAGCAACTGGAGTACGGTAGTGGCCCCGGTGATATTGAGCAAGAATTAGAACGTGCCAAAGAGAGCATAAGAAGATTGGAGCAG GAAGTCAATCAGGAAAGATCAAGAATGCAGTATTTACAGACTTTGCTAGAGAAAAGCGGCCCGGGCTCCCCTGAAGACAAGATCAGTCAACTGAAGGAAAAGATCC AGCAGTTGAAGCAAGAGAACCAGCAGCTGGAAGAGGAGAATTCTCAGCTCGAGTACGGTAGTGGACCAGGACTGGAAGAGGAATTGAAGCAACTGGAAGAAGAGCT ACAGGCTATCGAAGAGCAGTTAGCACAGCTCCAGTGGAAAGCCCAAGCTAGGAAAGAGAAATTGGCTCAGTTAAAAGAGAAGCTGAGTGGGCCTGGCTCCCCTGAG GACAAGAATTCTGAATTGAAGGAAGAAATCCAGCAACTTGAGGAAGAGAACCAGCAGCTAGAGGAGAAGATCTCAGAGCTGAAATACGGTTCCGGACCTGGTGATA TCGAACAAGAACTCGAGAGAGCAAAAGAATCTATCAGAAGGCTCGAACAAGAGGTTAATCAGGAAAGATCAAGGATGCAGTATCTTCAAACACTCCTGGAGAAATC CGGCCCGGGTAGCCCAGAGGATAAGAATAGTGAACTTAAAGAGGAGATCCAGCAGTTGGAAGAGGAGAACCAACAGTTAGAGGAGAAAATCTCGGAGCTCAAGTAC GGTTCTGGACCAGGATCTCCCGAAGACGAGAACTCACAACTTGAGGAGAAGATATCCCAGCTGAAACAAAAGAATTCCGAACTAAAGGAAGAAATACAGCAACTGG AGTACGGACTTGAGCATCATCATCATCATCACCATCATTAA
TET12SN(3CC)-NEG	CATATGTCCCCGGAAGACGAAAATAGTCAGTTAGAGGAGAAAATCTCACAGCTCAAACAGAAGAATTCGGAGTTAAAAGAGGAGATTCAACAGCTTGAATACGGGT CAGGTCCTGGCTCACCAGAGGACAAGAACTCTGAGTTAAAAGAGGAAATTCAACAACTCGAAGAGGAAAACCAACAGTTAGAGGAGAAAATCTCTGAACTGAAGTA TGGCAGTGGACCAGGATCTCCAGAGGACAAGAACAGTGAATTGAAGGAAGAAATCCAACAATTGGAAGAAGAGAATCAACAACTCGAGGAGAAGATTTCGGAATTA AAGTATGGATCTGGTCCGGGACTAGAAGAGGAACTGAAGCAGCTAGAGGAAGAGTTGCAGGCAATCGAGGAGCAACTCGCTCAACTACAATGGAAAGCCCAAGCCC GTAAAGAGAAACTCGCACAGTTGAAGGAAAAGCTGTCCGGACCTGGGTCACCAGAAGATGAGAATTCTCAGCTCGAGGAAAAGATCAGCCAGTTAAAACAAAAGAA CAGCGAGCTGAAAGAGGAAATCCAGCAGCTAGAATACGGATCTGGCCCTGGTGATATTGAGCAGGAGCTCGAAAGAGCAAAAGAGAGCATAAGAAGGTTAGAACAG GAAGTCAATCAGGAGCGATCTCGTATGCAATACCTGCAGACCTTGTTGGAGAAATCAGGTCCTGGATCACCAGAAGACAAGAACAGCGAGTTAAAAGAGGAGATTC AGCAGCTGGAAGAAGAGAATCAACAGTTGGAAGAGAAAATTTCAGAATTGAAGTATGGATCCGGACCCGGGCTAGAGGAAGAGTTAAAACAGCTTGAGGAAGAGTT GCAGGCAATTGAGGAACAATTGGCACAGCTTCAGTGGAAGGCTCAGGCTAGAAAGGAAAAGCTCGCCCAGCTCAAAGAGAAATTATCCGGGCCAGGTTCTCCTGAG GACGAGATACAGCAGCTCGAAGAGGAGATAAGCCAATTAGAACAGAAGAACTCCGAACTTAAAGAAAAGAACCAAGAGTTAAAGTACGGGAGTGGTCCAGGAGACA TCGAACAGGAACTGGAAAGAGCAAAAGAGTCTATAAGAAGATTAGAGCAAGAGGTAAACCAGGAGCGATCACGAATGCAGTACCTGCAAACCTTATTAGAAAAGTC GGGGCCGGGATCACCCGAGGATGAAAATTCTCAGCTTGAGGAAAAGATATCCCAATTGAAGCAAAAGAATTCCGAGCTGAAGGAAGAAATACAGCAGCTTGAGTAC GGATCCGGTCCAGGGTCTCCAGAGGACAAAATCAGTCAATTGAAGGAGAAAATCCAACAGTTAAAACAAGAGAACCAACAGCTTGAAGAGGAAAACAGTCAACTCG AATACGGACTAGAACATCATCATCACCATCATCATCATTAA

TET12SN(3CC)-P3:P4	CATATGTCTCCGGAAGATGAAAACAGCCAGTTAGAAGAAAAAATTAGTCAGCTGAAGCAGAAAAACTCGGAGCTGAAAGAAGAGATTCAGCAGCTGGAATACGGCT<br>CAGGTCCGGGTTCTCCTGAGGATAAAATCTCGCAGTTGAAAGAAAAAATTCAGCAACTGAAACAAGAGAATCAACAGCTGGAAGAAGAGAATTCGCAGCTGGAATA<br>CGGTTCAGGTCCGGGCTCACCGGAAGACGAGATCCAGCAGTTGGAAGAAGAAATCTCTCAGCTGGAGCAGAAAAACAGCGAACTGAAAGAAAAAAACCAGGAACTG<br>AAATATGGGTCTGGCCCGGGCCTGGAGGAGGAACTGAAGCAGCTGGAAGAAGAACTGCAGGCGATTGAAGAGCAACTGGCACAGTTACAGTGGAAGGCCCAGGCGC<br>GTAAAGAGAAATTAGCGCAGTTGAAAGAGAAGCTGGGTAGCGGTCCTGGCTCACCCGAAGATGAAATTCAGCAGCTGGAGGAGGAGATTAGCCAGTTAGAACAAAA<br>AAACTCCGAACTGAAAGAGAAAAATCAGGAACTTAAATATGGTTCGGGTCCGGGTGACATTGAGCAAGAGCTGGAACGCGCCAAAGAATCCATTCGTCGTTTGGAA<br>CAAGAAGTTAATCAGGAACGCTCACGTATGCAATATCTGCAGACCCTGCTGGAGAAAGGATCCGGCCCGGGTAGCCCCGAGGATAAAAATTCAGAACTGAAAGAAG<br>AAATTCAGCAACTGGAAGAAGAAAATCAACAATTAGAAGAGAAAATCTCGGAGCTGAAGTATGGTAGCGGTCCGGGCTTAGAGGAGGAGTTGAAACAGTTGGAAGA<br>AGAGCTTCAAGCAATCGAGGAGCAGTTAGCTCAATTGCAGTGGAAAGCTCAAGCCCGCAAGGAAAAGCTGGCGCAGCTGAAAGAGAAGCTGGGCAGTGGCCCGGGT<br>AGTCCTGAGGATAAGATTTCGCAGCTGAAGGAGAAGATTCAACAGTTGAAGCAAGAGAATCAACAATTAGAGGAGGAAAATTCACAGTTGGAGTATGGTAGCGGCC<br>CTGGTGACATCGAACAAGAACTTGAACGTGCCAAGGAATCAATTCGCCGTTTGGAACAAGAAGTGAATCAAGAACGTAGCCGCATGCAGTACTTACAGACACTGTT<br>GGAGAAGGGTAGCGGACCGGGCTCCCCTGAAGACAAGATTAGCCAATTAAAAGAGAAAATCCAACAATTGAAACAGGAAAATCAGCAGCTTGAGGAAGAAAATTCG<br>CAACTTGAATACGGTAGCGGTCCTGGTAGTCCGGAAGATGAAATCCAGCAATTAGAGGAGGAGATTTCTCAACTTGAGCAAAAGAACTCTGAGCTGAAAGAGAAGA<br>ATCAGGAGCTGAAATATCTCGAGCATCATCATCACCATCATCATCATTAA
TET12SN(3CC-NEG)-P3:P4	CATATGTCACCGGAAGACGAGATCCAGCAGTTGGAAGAAGAAATCTCTCAGCTGGAGCAGAAAAACAGCGAACTGAAAGAAAAAAACCAGGAACTGAAATATGGCT<br>CAGGTCCGGGTTCTCCTGAGGATAAAATCTCGCAGTTGAAAGAAAAAATTCAGCAACTGAAACAAGAGAATCAACAGCTGGAAGAAGAGAATTCGCAGCTGGAATA<br>CGGTTCAGGTCCGGGCAGTCCTGAGGATAAGATTTCGCAGCTGAAGGAGAAGATTCAACAGTTGAAGCAAGAGAATCAACAATTAGAGGAGGAAAATTCACAGTTG<br>GAGTATGGGTCTGGCCCGGGCCTGGAGGAGGAACTGAAGCAGCTGGAAGAAGAACTGCAGGCGATTGAAGAGCAACTGGCACAGTTACAGTGGAAGGCCCAGGCGC<br>GTAAAGAGAAATTAGCGCAGTTGAAAGAGAAGCTGGGTAGCGGTCCTGGCTCACCCGAAGATGAAATTCAGCAGCTGGAGGAGGAGATTAGCCAGTTAGAACAAAA<br>AAACTCCGAACTGAAAGAGAAAAATCAGGAACTTAAATATGGTTCGGGTCCGGGTGACATTGAGCAAGAGCTGGAACGCGCCAAAGAATCCATTCGTCGTTTGGAA<br>CAAGAAGTTAATCAGGAACGCTCACGTATGCAATATCTGCAGACCCTGCTGGAGAAAGGATCCGGCCCGGGTTCCCCTGAAGACAAGATTAGCCAATTAAAAGAGA<br>AAATCCAACAATTGAAACAGGAAAATCAGCAGCTTGAGGAAGAAAATTCGCAACTTGAATACGGTAGCGGTCCGGGCTTAGAGGAGGAGTTGAAACAGTTGGAAGA<br>AGAGCTTCAAGCAATCGAGGAGCAGTTAGCTCAATTGCAGTGGAAAGCTCAAGCCCGCAAGGAAAAGCTGGCGCAGCTGAAAGAGAAGCTGGGCAGTGGCCCGGGT<br>TCTCCGGAAGATGAAAACAGCCAGTTAGAAGAAAAAATTAGTCAGCTGAAGCAGAAAAACTCGGAGCTGAAAGAAGAGATTCAGCAGCTGGAATACGGTAGCGGCC<br>CTGGTGACATCGAACAAGAACTTGAACGTGCCAAGGAATCAATTCGCCGTTTGGAACAAGAAGTGAATCAAGAACGTAGCCGCATGCAGTACTTACAGACACTGTT<br>GGAGAAGGGTAGCGGACCGGGCAGTCCGGAAGATGAAATCCAGCAATTAGAGGAGGAGATTTCTCAACTTGAGCAAAAGAACTCTGAGCTGAAAGAGAAGAATCAG<br>GAGCTGAAATATGGTAGCGGTCCTGGTAGCCCCGAGGATAAAAATTCAGAACTGAAAGAAGAAATTCAGCAACTGGAAGAAGAAAATCAACAATTAGAAGAGAAAA<br>TCTCGGAGCTGAAGTATCTCGAGCATCATCATCACCATCATCATCATTAA
TET12SN-GCNcys	CATATGCTTGAGGAAGAACTGAAGCAGTTGGAAGAAGAGTTGCAAGCGATCGAAGAGCAGTTGGCGCAGCTGCAGTGGAAGGCACAGGCGCGCAAAGAGAAGTTAG<br>CGCAGTTAAAAGAAAAGTTGTCTGGGCCTGGCTCACCGGAAGACGAGATCCAGCAGTTGGAAGAAGAAATCTCTCAGCTGGAGCAGAAAAACAGCGAACTGAAAGA<br>AAAAAACCAGGAACTGAAATATGGCAGCGGCCCGGGTGATATTGAGCAGGAACTGGAACGTGCCAAAGAATCGATTCGTCGGTTAGAACAGGAGGTTAACCAAGAA<br>CGCTCGCGCATGCAATACTTGCAAACTTTGCTTGAAAAATCCGGTCCTGGACAACTGGAGGACAAGGTGGAAGAATTGCTCTCCAAAAACTATCACTTGGAAAACG<br>AAGTTGAGCGCCTGAAGAAACTGGTAGGTTGTGGCCCGGGGCTGGAGGAGGAACTGAAGCAGCTGGAAGAAGAACTGCAGGCGATTGAAGAGCAACTGGCACAGTT<br>ACAGTGGAAGGCCCAGGCGCGTAAAGAGAAATTAGCGCAGTTGAAAGAGAAGCTGTCAGGGCCAGGAAGCCCGGAAGATGAAATCCAGCAACTTGAAGAGAAAAAT<br>TCACAACTCAAGCAGGAAATTTCCCAGCTTGAAGAAAAAAAATCAGGAATTAAAATATGGATCTGGCCCCGGTCAACTGGAAGACAAGGTCGAAGAGCTTTTGTCCA<br>AAAACTACCATTTAGAAAATGAAGTGGAACGCCTTAAAAAACTGGTAGGTTGCGGACCGGGTTCTCCTGAGGATAAAATCTCGCAGTTGAAAGAAAAAATTCAGCA<br>ACTGAAACAAGAGAATCAACAGCTGGAAGAAGAGAATTCGCAGCTGGAATACGGCTCAGGTCCTGGTTCTCCGGAAGATGAAAACAGCCAGTTAGAAGAAAAAATT<br>AGTCAGCTGAAGCAGAAAAACTCGGAGCTGAAAGAAGAGATTCAGCAGCTGGAATACGGCTCCGGCCCGGGGTCCCCTGAAGATAAAATTTCCGAGCTGAAAGAAG<br>AAAATCAGCAGCTGGAACAGAAGATTCAACAACTGAAAGAAGAAAACTCGCAACTGGAATATGGCTCGGGACCGGGGGACATTGAGCAAGAGCTGGAACGCGCCAA<br>AGAATCCATTCGTCGTTTGGAACAAGAAGTTAATCAGGAACGCTCACGTATGCAATATCTGCAGACCCTGCTGGAGAAATCCGGTCCAGGTAGCCCCGAGGATAAA<br>AATTCAGAACTGAAAGAAGAAATTCAGCAACTGGAAGAAGAAAATCAACAATTAGAAGAGAAAATCTCGGAGCTGAAGTATGGCCTCGAGCACCACCACCACCACC<br>ACCACCACTAATTGATTAA

TET12SN-P7P8cys	CATATGCTTGAGGAAGAACTGAAGCAGTTGGAAGAAGAGTTGCAAGCGATCGAAGAGCAGTTGGCGCAGCTGCAGTGGAAGGCACAGGCGCGCAAAGAGAAGTTAG<br>CGCAGTTAAAAGAAAAGTTGTCTGGGCCTGGCTCACCGGAAGACGAGATCCAGCAGTTGGAAGAAGAAATCTCTCAGCTGGAGCAGAAAAACAGCGAACTGAAAGA<br>AAAAAACCAGGAACTGAAATATGGCAGCGGCCCGGGTGATATTGAGCAGGAACTGGAACGTGCCAAAGAATCGATTCGTCGGTTAGAACAGGAGGTTAACCAAGAA<br>CGCTCGCGCATGCAATACTTGCAAACTTTGCTTGAAAAATCCGGTCCTGGACAACTGGAGGACAAGGTGGAAGAATTGCTCTCCAAAAACTATCACTTGGAAAACG<br>AAGTTGAGCGCCTGAAGAAACTGGTAGGTTCAGGCCCGGGGCTGGAGGAGGAACTGAAGCAGCTGGAAGAAGAACTGCAGGCGATTGAAGAGCAACTGGCACAGTT<br>ACAGTGGAAGGCCCAGGCGCGTAAAGAGAAATTAGCGCAGTTGAAAGAGAAGCTGTCAGGGCCAGGAAGCCCGGAAGATGAAATCCAGCAACTTGAAGAGAAAAAT<br>TCACAACTCAAGCAGGAAATTTCCCAGCTTGAAGAAAAAAAATCAGGAATTAAAATATGGATGTGGCCCCGGTCAACTGGAAGACAAGGTCGAAGAGCTTTTGTCCA<br>AAAACTACCATTTAGAAAATGAAGTGGAACGCCTTAAAAAACTGGTAGGTAGCGGACCGGGTTCTCCTGAGGATAAAATCTCGCAGTTGAAAGAAAAAATTCAGCA<br>ACTGAAACAAGAGAATCAACAGCTGGAAGAAGAGAATTCGCAGCTGGAATACGGCTCAGGTCCTGGTTCTCCGGAAGATGAAAACAGCCAGTTAGAAGAAAAAATT<br>AGTCAGCTGAAGCAGAAAAACTCGGAGCTGAAAGAAGAGATTCAGCAGCTGGAATACGGCTCCGGCCCGGGGTCCCCTGAAGATAAAATTTCCGAGCTGAAAGAAG<br>AAAATCAGCAGCTGGAACAGAAGATTCAACAACTGAAAGAAGAAAACTCGCAACTGGAATATGGCTGTGGACCGGGGGACATTGAGCAAGAGCTGGAACGCGCCAA<br>AGAATCCATTCGTCGTTTGGAACAAGAAGTTAATCAGGAACGCTCACGTATGCAATATCTGCAGACCCTGCTGGAGAAATCCGGTCCAGGTAGCCCCGAGGATAAA<br>AATTCAGAACTGAAAGAAGAAATTCAGCAACTGGAAGAAGAAAATCAACAATTAGAAGAGAAAATCTCGGAGCTGAAGTATGGCCTCGAGCACCACCACCACCACC<br>ACCACCACTAATTGATTAA
TET12SN-P5P6cys	CATATGCTTGAGGAAGAACTGAAGCAGTTGGAAGAAGAGTTGCAAGCGATCGAAGAGCAGTTGGCGCAGCTGCAGTGGAAGGCACAGGCGCGCAAAGAGAAGTTAG<br>CGCAGTTAAAAGAAAAGTTGTCTGGGCCTGGCTCACCGGAAGACGAGATCCAGCAGTTGGAAGAAGAAATCTCTCAGCTGGAGCAGAAAAACAGCGAACTGAAAGA<br>AAAAAACCAGGAACTGAAATATGGCAGCGGCCCGGGTGATATTGAGCAGGAACTGGAACGTGCCAAAGAATCGATTCGTCGGTTAGAACAGGAGGTTAACCAAGAA<br>CGCTCGCGCATGCAATACTTGCAAACTTTGCTTGAAAAATCCGGTCCTGGACAACTGGAGGACAAGGTGGAAGAATTGCTCTCCAAAAACTATCACTTGGAAAACG<br>AAGTTGAGCGCCTGAAGAAACTGGTAGGTTCAGGCCCGGGGCTGGAGGAGGAACTGAAGCAGCTGGAAGAAGAACTGCAGGCGATTGAAGAGCAACTGGCACAGTT<br>ACAGTGGAAGGCCCAGGCGCGTAAAGAGAAATTAGCGCAGTTGAAAGAGAAGCTGTCAGGGCCAGGAAGCCCGGAAGATGAAATCCAGCAACTTGAAGAGAAAAAT<br>TCACAACTCAAGCAGGAAATTTCCCAGCTTGAAGAAAAAAAATCAGGAATTAAAATATGGATCTGGCCCCGGTCAACTGGAAGACAAGGTCGAAGAGCTTTTGTCCA<br>AAAACTACCATTTAGAAAATGAAGTGGAACGCCTTAAAAAACTGGTAGGTAGCGGACCGGGTTCTCCTGAGGATAAAATCTCGCAGTTGAAAGAAAAAATTCAGCA<br>ACTGAAACAAGAGAATCAACAGCTGGAAGAAGAGAATTCGCAGCTGGAATACGGCTCAGGTCCTGGTTCTCCGGAAGATGAAAACAGCCAGTTAGAAGAAAAAATT<br>AGTCAGCTGAAGCAGAAAAACTCGGAGCTGAAAGAAGAGATTCAGCAGCTGGAATACGGCTGCGGCCCGGGGTCCCCTGAAGATAAAATTTCCGAGCTGAAAGAAG<br>AAAATCAGCAGCTGGAACAGAAGATTCAACAACTGAAAGAAGAAAACTCGCAACTGGAATATGGCTCGGGACCGGGGGACATTGAGCAAGAGCTGGAACGCGCCAA<br>AGAATCCATTCGTCGTTTGGAACAAGAAGTTAATCAGGAACGCTCACGTATGCAATATCTGCAGACCCTGCTGGAGAAATCCGGTCCAGGTAGCCCCGAGGATAAA<br>AATTCAGAACTGAAAGAAGAAATTCAGCAACTGGAAGAAGAAAATCAACAATTAGAAGAGAAAATCTCGGAGCTGAAGTATGGCTGCCTCGAGCACCACCACCACC<br>ACCACCACCACTAA
TET12SN-BCRcys	CATATGCTTGAGGAAGAACTGAAGCAGTTGGAAGAAGAGTTGCAAGCGATCGAAGAGCAGTTGGCGCAGCTGCAGTGGAAGGCACAGGCGCGCAAAGAGAAGTTAG<br>CGCAGTTAAAAGAAAAGTTGTCTGGGCCTGGCTCACCGGAAGACGAGATCCAGCAGTTGGAAGAAGAAATCTCTCAGCTGGAGCAGAAAAACAGCGAACTGAAAGA<br>AAAAAACCAGGAACTGAAATATGGCAGCGGCCCGGGTGATATTGAGCAGGAACTGGAACGTGCCAAAGAATCGATTCGTTGCTTAGAACAGGAGGTTAACCAAGAA<br>CGCTCGCGCATGCAATACTTGCAAACTTTGCTTGAAAAATCCGGTCCTGGACAACTGGAGGACAAGGTGGAAGAATTGCTCTCCAAAAACTATCACTTGGAAAACG<br>AAGTTGAGCGCCTGAAGAAACTGGTAGGTTCAGGCCCGGGGCTGGAGGAGGAACTGAAGCAGCTGGAAGAAGAACTGCAGGCGATTGAAGAGCAACTGGCACAGTT<br>ACAGTGGAAGGCCCAGGCGCGTAAAGAGAAATTAGCGCAGTTGAAAGAGAAGCTGTCAGGGCCAGGAAGCCCGGAAGATGAAATCCAGCAACTTGAAGAGAAAAAT<br>TCACAACTCAAGCAGGAAATTTCCCAGCTTGAAGAAAAAAAATCAGGAATTAAAATATGGATCTGGCCCCGGTCAACTGGAAGACAAGGTCGAAGAGCTTTTGTCCA<br>AAAACTACCATTTAGAAAATGAAGTGGAACGCCTTAAAAAACTGGTAGGTAGCGGACCGGGTTCTCCTGAGGATAAAATCTCGCAGTTGAAAGAAAAAATTCAGCA<br>ACTGAAACAAGAGAATCAACAGCTGGAAGAAGAGAATTCGCAGCTGGAATACGGCTCAGGTCCTGGTTCTCCGGAAGATGAAAACAGCCAGTTAGAAGAAAAAATT<br>AGTCAGCTGAAGCAGAAAAACTCGGAGCTGAAAGAAGAGATTCAGCAGCTGGAATACGGCTCCGGCCCGGGGTCCCCTGAAGATAAAATTTCCGAGCTGAAAGAAG<br>AAAATCAGCAGCTGGAACAGAAGATTCAACAACTGAAAGAAGAAAACTCGCAACTGGAATATGGCTCGGGACCGGGGGACATTGAGCAAGAGCTGGAACGCGCCAA<br>AGAATCCATTCGTTGTTTGGAACAAGAAGTTAATCAGGAACGCTCACGTATGCAATATCTGCAGACCCTGCTGGAGAAATCCGGTCCAGGTAGCCCCGAGGATAAA<br>AATTCAGAACTGAAAGAAGAAATTCAGCAACTGGAAGAAGAAAATCAACAATTAGAAGAGAAAATCTCGGAGCTGAAGTATGGCCTCGAGCACCACCACCACCACC<br>ACCACCACTAATTGATTAA

TET12SN-P3P4cys	CATATGCTTGAGGAAGAACTGAAGCAGTTGGAAGAAGAGTTGCAAGCGATCGAAGAGCAGTTGGCGCAGCTGCAGTGGAAGGCACAGGCGCGCAAAGAGAAGTTAG<br>CGCAGTTAAAAGAAAAGTTGTCTGGGCCTGGCTCACCGGAAGACGAGATCCAGCAGTTGGAAGAAGAAATCTCTCAGCTGGAGCAGAAAAACAGCGAACTGAAAGA<br>AAAAAACCAGGAACTGAAATATGGCTGCGGCCCGGGTGATATTGAGCAGGAACTGGAACGTGCCAAAGAATCGATTCGTCGGTTAGAACAGGAGGTTAACCAAGAA<br>CGCTCGCGCATGCAATACTTGCAAACTTTGCTTGAAAAATCCGGTCCTGGACAACTGGAGGACAAGGTGGAAGAATTGCTCTCCAAAAACTATCACTTGGAAAACG<br>AAGTTGAGCGCCTGAAGAAACTGGTAGGTTCAGGCCCGGGGCTGGAGGAGGAACTGAAGCAGCTGGAAGAAGAACTGCAGGCGATTGAAGAGCAACTGGCACAGTT<br>ACAGTGGAAGGCCCAGGCGCGTAAAGAGAAATTAGCGCAGTTGAAAGAGAAGCTGTCAGGGCCAGGAAGCCCGGAAGATGAAATCCAGCAACTTGAAGAGAAAAAT<br>TCACAACTCAAGCAGGAAATTTCCCAGCTTGAAGAAAAAAAATCAGGAATTAAAATATGGATCTGGCCCCGGTCAACTGGAAGACAAGGTCGAAGAGCTTTTGTCCA<br>AAAACTACCATTTAGAAAATGAAGTGGAACGCCTTAAAAAACTGGTAGGTAGCGGACCGGGTTCTCCTGAGGATAAAATCTCGCAGTTGAAAGAAAAAATTCAGCA<br>ACTGAAACAAGAGAATCAACAGCTGGAAGAAGAGAATTCGCAGCTGGAATACGGCTGCGGTCCTGGTTCTCCGGAAGATGAAAACAGCCAGTTAGAAGAAAAAATT<br>AGTCAGCTGAAGCAGAAAAACTCGGAGCTGAAAGAAGAGATTCAGCAGCTGGAATACGGCTCCGGCCCGGGGTCCCCTGAAGATAAAATTTCCGAGCTGAAAGAAG<br>AAAATCAGCAGCTGGAACAGAAGATTCAACAACTGAAAGAAGAAAACTCGCAACTGGAATATGGCTCGGGACCGGGGGACATTGAGCAAGAGCTGGAACGCGCCAA<br>AGAATCCATTCGTCGTTTGGAACAAGAAGTTAATCAGGAACGCTCACGTATGCAATATCTGCAGACCCTGCTGGAGAAATCCGGTCCAGGTAGCCCCGAGGATAAA<br>AATTCAGAACTGAAAGAAGAAATTCAGCAACTGGAAGAAGAAAATCAACAATTAGAAGAGAAAATCTCGGAGCTGAAGTATGGCCTCGAGCACCACCACCACCACC<br>ACCACCACTAATTGATTAA
TET12SN-APHcys	CATATGCTTGAGGAAGAACTGAAGCAGTTGGAAGAAGAGTTGCAAGCGATCGAAGAGCAGTTGGCGTGCCTGCAGTGGAAGGCACAGGCGCGCAAAGAGAAGTTAG<br>CGCAGTTAAAAGAAAAGTTGTCTGGGCCTGGCTCACCGGAAGACGAGATCCAGCAGTTGGAAGAAGAAATCTCTCAGCTGGAGCAGAAAAACAGCGAACTGAAAGA<br>AAAAAACCAGGAACTGAAATATGGCAGCGGCCCGGGTGATATTGAGCAGGAACTGGAACGTGCCAAAGAATCGATTCGTCGGTTAGAACAGGAGGTTAACCAAGAA<br>CGCTCGCGCATGCAATACTTGCAAACTTTGCTTGAAAAATCCGGTCCTGGACAACTGGAGGACAAGGTGGAAGAATTGCTCTCCAAAAACTATCACTTGGAAAACG<br>AAGTTGAGCGCCTGAAGAAACTGGTAGGTTCAGGCCCGGGGCTGGAGGAGGAACTGAAGCAGCTGGAAGAAGAACTGCAGGCGATTGAAGAGCAACTGGCATGCTT<br>ACAGTGGAAGGCCCAGGCGCGTAAAGAGAAATTAGCGCAGTTGAAAGAGAAGCTGTCAGGGCCAGGAAGCCCGGAAGATGAAATCCAGCAACTTGAAGAGAAAAAT<br>TCACAACTCAAGCAGGAAATTTCCCAGCTTGAAGAAAAAAAATCAGGAATTAAAATATGGATCTGGCCCCGGTCAACTGGAAGACAAGGTCGAAGAGCTTTTGTCCA<br>AAAACTACCATTTAGAAAATGAAGTGGAACGCCTTAAAAAACTGGTAGGTAGCGGACCGGGTTCTCCTGAGGATAAAATCTCGCAGTTGAAAGAAAAAATTCAGCA<br>ACTGAAACAAGAGAATCAACAGCTGGAAGAAGAGAATTCGCAGCTGGAATACGGCTCAGGTCCTGGTTCTCCGGAAGATGAAAACAGCCAGTTAGAAGAAAAAATT<br>AGTCAGCTGAAGCAGAAAAACTCGGAGCTGAAAGAAGAGATTCAGCAGCTGGAATACGGCTCCGGCCCGGGGTCCCCTGAAGATAAAATTTCCGAGCTGAAAGAAG<br>AAAATCAGCAGCTGGAACAGAAGATTCAACAACTGAAAGAAGAAAACTCGCAACTGGAATATGGCTCGGGACCGGGGGACATTGAGCAAGAGCTGGAACGCGCCAA<br>AGAATCCATTCGTCGTTTGGAACAAGAAGTTAATCAGGAACGCTCACGTATGCAATATCTGCAGACCCTGCTGGAGAAATCCGGTCCAGGTAGCCCCGAGGATAAA<br>AATTCAGAACTGAAAGAAGAAATTCAGCAACTGGAAGAAGAAAATCAACAATTAGAAGAGAAAATCTCGGAGCTGAAGTATGGCCTCGAGCACCACCACCACCACC<br>ACCACCACTAATTGATTAA

Supplementary Table 11. DNA sequences of characterised CCPO tetrahedra.

Name	DNA sequence
Pos1_APH	GGTCTCCCACGCATATGCTTGAGGAAGAACTGAAGCAGTTGGAAGAAGAGTTGCAAGCGATCGAAGAGCAGTTGGCGCAGCTGCAGTGGAAGGCACAGGCGCGCAAAGAGAAGTTAGCGCAGTTAAAAGAAAAGTTGGGCTCACGAGACC
Pos1_P3	GGTCTCCCACGCATATGTCACCGGAAGACGAGATCCAGCAGTTGGAAGAAGAAATCTCTCAGCTGGAGCAGAAAAACAGCGAACTGAAAGAAAAAAACCAGGAACTGAAATATGGCTCACGAGACC
Pos1_P5	GGTCTCCCACGCATATGTCTCCGGAAGATGAAAACAGCCAGTTAGAAGAAAAAATTAGTCAGCTGAAGCAGAAAAACTCGGAGCTGAAAGAAGAGATTCAGCAGCTGGAATACGGCTCACGAGACC
Pos2_P3	GGTCTCCCTCAGGTCCGGGTTCACCGGAAGACGAGATCCAGCAGTTGGAAGAAGAAATCTCTCAGCTGGAGCAGAAAAACAGCGAACTGAAAGAAAAAAACCAGGAACTGAAATATGGTTCAGCGAGACC
Pos2_P4	GGTCTCCCTCAGGTCCGGGTTCTCCTGAGGATAAAATCTCGCAGTTGAAAGAAAAAATTCAGCAACTGAAACAAGAGAATCAACAGCTGGAAGAAGAGAATTCGCAGCTGGAATACGGTTCAGCGAGACC
Pos2_P5	GGTCTCCCTCAGGTCCGGGTTCGCCAGAGGATGAGAATAGCCAACTTGAAGAAAAGATCAGTCAATTGAAGCAAAAAAACAGTGAACTGAAGGAGGAAATTCAACAGTTAGAGTACGGTTCAGCGAGACC
Pos3_APH	GGTCTCCTCAGGTCCGGGCTTAGAGGAGGAGTTGAAACAGTTGGAAGAAGAGCTTCAAGCAATCGAGGAGCAGTTAGCTCAATTGCAGTGGAAAGCTCAAGCCCGCAAGGAAAAGCTGGCGCAGCTGAAAGAGAAGCTGGGGTCGAGACC
Pos3_BCR	GGTCTCCTCAGGTCCGGGCGATATTGAGCAGGAACTGGAACGTGCCAAAGAATCGATTCGTCGGTTAGAACAGGAGGTTAACCAAGAACGCTCGCGCATGCAATACTTGCAAACTTTGCTTGAAAAAGGGTCGAGACC
Pos3_P3	GGTCTCCTCAGGTCCGGGCTCACCGGAAGACGAGATCCAGCAGTTGGAAGAAGAAATCTCTCAGCTGGAGCAGAAAAACAGCGAACTGAAAGAAAAAAACCAGGAACTGAAATATGGGTCGAGACC
Pos3_P4	GGTCTCCTCAGGTCCGGGCAGTCCTGAGGATAAGATTTCGCAGCTGAAGGAGAAGATTCAACAGTTGAAGCAAGAGAATCAACAATTAGAGGAGGAAAATTCACAGTTGGAGTATGGGTCGAGACC
Pos4_APH	GGTCTCCGGGTCTGGCCCGGGCCTGGAGGAGGAACTGAAGCAGCTGGAAGAAGAACTGCAGGCGATTGAAGAGCAACTGGCACAGTTACAGTGGAAGGCCCAGGCGCGTAAAGAGAAATTAGCGCAGTTGAAAGAGAAGCTGGGTAGCGGTCCTGCGAGACC
Pos4_GCN	GGTCTCCGGGTCTGGCCCGGGCCAACTGGAGGACAAGGTGGAAGAATTGCTCTCCAAAAACTATCACTTGGAAAACGAAGTTGAGCGCCTGAAGAAACTGGTAGGTAGCGTTCCTGCGAGACC
Pos4_P3	GGTCTCCGGGTCTGGCCCGGGCTCACCGGAAGACGAGATCCAGCAGTTGGAAGAAGAAATCTCTCAGCTGGAGCAGAAAAACAGCGAACTGAAAGAAAAAAAACCAGGAACTGAAATATGGTAGCGGTCCTGCGAGACC
Pos5_APH	GGTCTCCCCTGGCCTGGAGGAGGAACTGAAGCAGCTGGAAGAAGAACTGCAGGCGATTGAAGAGCAACTGGCACAGTTACAGTGGAAGGCCCAGGCGCGTAAAGAGAAATTAGCGCAGTTGAAAGAGAAGCTGGGTTCGCGAGACC
Pos5_P3	GGTCTCCCCTGGCTCACCCGAAGATGAAATTCAGCAGCTGGAGGAGGAGATTAGCCAGTTAGAACAAAAAAACTCCGAACTGAAAGAGAAAAATCAGGAACTTAAATATGGTTCGCGAGACC
Pos6_BCR	GGTCTCCTTCGGGTCCGGGTGACATTGAGCAAGAGCTGGAACGCGCCAAAGAATCCATTCGTCGTTTGGAACAAGAAGTTAATCAGGAACGCTCACGTATGCAATATCTGCAGACCCTGCTGGAGAAAGGATCCCGGCGCTCGCTCGAGCGAGACC
Pos6_P3	GGTCTCCTTCGGGTCCGGGTTCACCCGAAGATGAAATTCAGCAGCTGGAGGAGGAGATTAGCCAGTTAGAACAAAAAAACTCCGAACTGAAAGAGAAAAATCAGGAACTTAAATATGGATCCCGGCGCTCGCTCGAGCGAGACC
Pos6_P7	GGTCTCCTTCGGGTCCGGGTAGCCCGGAAGATGAAATCCAGCAACTTGAAGAGAAAAATTCACAACTCAAGCAGGAAATTTCCCAGCTTGAAGAAAAAAATCAGGAATTAAAATATGGATCCCGGCGCTCGCTCGAGCGAGACC
Pos7_GCN	GGTCTCCCACGGATCCGGCCCGGGTCAACTGGAAGACAAGGTCGAAGAGCTTTTGTCCAAAAACTACCATTTAGAAAATGAAGTGGAACGCCTTAAAAAACTGGTAGGTAGCGAGACC
Pos7_P4	GGTCTCCCACGGATCCGGCCCGGGTTCTCCTGAGGATAAAATCTCGCAGTTGAAAGAAAAAATTCAGCAACTGAAACAAGAGAATCAACAGCTGGAAGAAGAGAATTCGCAGCTGGAATACGGTAGCGAGACC

Pos7_P6	GGTCTCCCACGGATCCGGCCCGGGTAGCCCCGAGGATAAAAATTCAGAACTGAAAGAAGAAATTCAGCAACTGGAAGAAGAAAATCAACAATTAGAAGAGAAAATCTCGGAGCTGAAGTATGGTAGCGAGACC
Pos8_APH	GGTCTCCGTAGCGGTCCGGGCTTAGAGGAGGAGTTGAAACAGTTGGAAGAAGAGCTTCAAGCAATCGAGGAGCAGTTAGCTCAATTGCAGTGGAAAGCTCAAGCCCGCAAGGAAAAGCTGGCGCAGCTGAAAGAGAAGCTGGGCAGTCGAGACC
Pos8_P4	GGTCTCCGTAGCGGTCCGGGCTCTCCTGAGGATAAAATCTCGCAGTTGAAAGAAAAAATTCAGCAACTGAAACAAGAGAATCAACAGCTGGAAGAAGAGAATTCGCAGCTGGAATACGGCAGTCGAGACC
Pos8_P6	GGTCTCCGTAGCGGTCCGGGCTCTCCGGAAGACAAGAATTCTGAGCTGAAGGAAGAAATTCAACAATTAGAAGAGGAGAACCAACAACTGGAGGAGAAAATTTCAGAGCTGAAATATGGCAGTCGAGACC
Pos9_P4	GGTCTCCCAGTGGCCCGGGTAGTCCTGAGGATAAGATTTCGCAGCTGAAGGAGAAGATTCAACAGTTGAAGCAAGAGAATCAACAATTAGAGGAGGAAAATTCACAGTTGGAGTATGGTAGCGGCCCTCGAGACC
Pos9_P5	GGTCTCCCAGTGGCCCGGGTTCTCCGGAAGATGAAAACAGCCAGTTAGAAGAAAAAATTAGTCAGCTGAAGCAGAAAAACTCGGAGCTGAAAGAAGAGATTCAGCAGCTGGAATACGGTAGCGGCCCTCGAGACC
Pos10_BCR	GGTCTCCCCCTGGTGACATCGAACAAGAACTTGAACGTGCCAAGGAATCAATTCGCCGTTTGGAACAAGAAGTGAATCAAGAACGTAGCCGCATGCAGTACTTACAGACACTGTTGGAGAAGGGTAGCGGACCGCGAGACC
Pos10_P4	GGTCTCCCCCTGGTAGTCCTGAGGATAAGATTTCGCAGCTGAAGGAGAAGATTCAACAGTTGAAGCAAGAGAATCAACAATTAGAGGAGGAAAATTCACAGTTGGAGTATGGTAGCGGACCGCGAGACC
Pos10_P8	GGTCTCCCCCTGGTTCCCCTGAAGATAAAATTTCCGAGCTGAAAGAAGAAAATCAGCAGCTGGAACAGAAGATTCAACAACTGAAAGAAGAAAACTCGCAACTGGAATATGGTAGCGGACCGCGAGACC
Pos11_APH	GGTCTCCACCGGGCCTGGAAGAGGAGCTGAAGCAATTGGAAGAAGAATTACAGGCGATTGAGGAGCAATTGGCACAGCTTCAGTGGAAAGCTCAAGCACGCAAGGAAAAAATTAGCCCAACTTAAAGAGAAGTTAGGTACGAGACC
Pos11_BCR	GGTCTCCACCGGGCGACATTGAGCAAGAGCTGGAACGCGCCAAAGAATCCATTCGTCGTTTGGAACAAGAAGTTAATCAGGAACGCTCACGTATGCAATATCTGCAGACCCTGCTGGAGAAAGGTACGAGACC
Pos11_P3	GGTCTCCACCGGGCAGTCCGGAAGATGAAATCCAGCAATTAGAGGAGGAGATTTCTCAACTTGAGCAAAAGAACTCTGAGCTGAAAGAGAAGAATCAGGAGCTGAAATATGGTACGAGACC
Pos11_P4	GGTCTCCACCGGGCTCCCCTGAAGACAAGATTAGCCAATTAAAAGAGAAAATCCAACAATTGAAACAGGAAAATCAGCAGCTTGAGGAAGAAAATTCGCAACTTGAATACGGTACGAGACC
Pos12_P3	GGTCTCCGGTAGCGGTCCTGGTAGTCCGGAAGATGAAATCCAGCAATTAGAGGAGGAGATTTCTCAACTTGAGCAAAAGAACTCTGAGCTGAAAGAGAAGAATCAGGAGCTGAAATATCTCGAGCGAGACC
Pos12_P6	GGTCTCCGGTAGCGGTCCTGGTAGCCCCGAGGATAAAAATTCAGAACTGAAAGAAGAAATTCAGCAACTGGAAGAAGAAAATCAACAATTAGAAGAGAAAATCTCGGAGCTGAAGTATCTCGAGCGAGACC

Supplementary Table 12. DNA fragments used for cloning the DNA coding for the designs TET12SN(2CC), TET12SN(222CC), TET12SN(3CC)-P3:P4 and TET12SN(3CC-neg)-P3:P4 with Golden Gate Assembly.

Name	DNA sequence
F P3P4 cys	GAAATATGGCTGCGGCCCGGGTGATATTG
R P3P4 cys	GAGAACCAGGACCGCAGCCGTATTCCAG
F (pET)P3P4 cys	CTGGAATACGGCTGCGGTCCTGGTTCTC
R (pET)P3P4 cys	CAATATCACCCGGGCCGCAGCCATATTTC
F APH cys	GCAAGCGATCGAAGAGCAGTTGGCGTGCCTGCAGTGGAAGGCACAGGCGCGC
R APH cys	CGCGCCTGGGCCTTCCACTGTAAGCATGCCAGTTGCTCTTCAATCGCCTG
F APH(pET) cys	CAGGCGATTGAAGAGCAACTGGCATGCTTACAGTGGAAGGCCCAGGCGCG
R APH(pET) cys	GCGCGCCTGTGCCTTCCACTGCAGGCACGCCAACTGCTCTTCGATCGCTTGC
R BCR cys	GTTCCTGATTAACTTCTTGTTCCAAACAACGAATGGATTCTTTGGCGCGTTCC
F (pET)BCR cys	GGAACGCGCCAAAGAATCCATTCGTTGTTTGGAACAAGAAGTTAATCAGGAAC
R (pET)BCR cys	GTTCTTGGTTAACCTCCTGTTCTAAGCAACGAATCGATTCTTTGGCACGTTC
F GCN cys	GAGCGCCTGAAGAAACTGGTAGGTTGTGGCCCGGGGCTGGAGGAGGAACTG
R GCN cys	GATTTTATCCTCAGGAGAACCCGGTCCGCAACCTACCAGTTTTTTAAGGCGTTCC
F (pET) GCN cys	GGAACGCCTTAAAAAACTGGTAGGTTGCGGACCGGGTTCTCCTGAGGATAAAATC
R (pET) GCN cys	CAGTTCCTCCTCCAGCCCCGGGCCACAACCTACCAGTTTCTTCAGGCGCTC
F P7P8 cys	GAAAAAAATCAGGAATTAAAATATGGATGTGGCCCCGGTCAACTGGAAGACAAGG
R P7P8 cys	CTCTTGCTCAATGTCCCCCGGTCCACAGCCATATTCCAGTTGCGAGTTTTC
F (pET)P7P8 cys	GAAAACTCGCAACTGGAATATGGCTGTGGACCGGGGGACATTGAGCAAGAG
R (pET) P7P8 cys	CCTTGTCTTCCAGTTGACCGGGGCCACATCCATATTTTAATTCCTGATTTTTTTC
F P5P6 cys	GAGATTCAGCAGCTGGAATACGGCTGCGGCCCGGGGTCCCCTGAAGATAAAATTTC
R P5P6 cys	GTGGTGCTCGAGGCAGCCATACTTCAGCTCCGAGATTTTCTCTTCTAATTGTTGATTTTC
F (pET)P5P6 cys	GAGAAAATCTCGGAGCTGAAGTATGGCTGCCTCGAGCACCACCACCACCACCACCAC
R (pET) P5P6 cys	GAAATTTTATCTTCAGGGGACCCCGGGCCGCAGCCGTATTCCAGCTGCTGAATCTC

Supplementary Table 13. DNA primers used to introduce cysteine residues in the protein TET12SN at different positions. The primer pairs were used to amplify either the insert or the vector (pET) and then assembled via Gibson Assembly.

a)

time (min)	Phase B (%)
0	0
5	0
20	30
31	80
35	80
35.1	0
40	0

b)

time (min)	Phase B (%)
0	0
5	0
15	10
40	50
41	80
45	80
45.1	0
50	0

Supplementary Table 14. Gradients applied during HPLC purification of peptide-dye conjugates. (a) Gradient for the buffer system with phase A: 5 % ACN, 95 % H_2O, 0.1 % TFA, and phase B: 95 % ACN, 5 % H_2O, 0.1 % TFA. (b) Gradient for the buffer system with phase A: 5 % ACN, 95 % 0.1 M TEAA pH = 7, and phase B: 95 % ACN, 5 % H_2O.

Supplementary References

1. Gurnon, D. G., Whitaker, J. A. & Oakley, M. G. Design and characterization of a homodimeric antiparallel coiled coil. *J. Am. Chem. Soc.* **125,** 7518–7519 (2003).

2. Taylor, C. M. & Keating, A. E. Orientation and oligomerization specificity of the Bcr coiled-coil oligomerization domain. *Biochemistry* **44,** 16246–16256 (2005).

3. O'Shea, E. K., Rutkowski, R. & Kim, P. S. Evidence that the leucine zipper is a coiled coil. *Science* **243,** 538–542 (1989).

4. Zhu, H., Celinski, S. A., Scholtz, J. M. & Hu, J. C. The contribution of buried polar groups to the conformational stability of the GCN4 coiled coil. *J. Mol. Biol.* **300,** 1377–1387 (2000).

5. Crooks, R. O., Lathbridge, A., Panek, A. S. & Mason, J. M. Computational Prediction and Design for Creating Iteratively Larger Heterospecific Coiled Coil Sets. *Biochemistry* **56,** 1573–1584 (2017).

6. Gianni, S., Ivarsson, Y., Jemth, P., Brunori, M. & Travaglini-Allocatelli, C. Identification and characterization of protein folding intermediates. *Biophysical Chemistry* **128,** 105–113 (2007).

7. Ljubetič, A. *et al.* Design of coiled-coil protein-origami cages that self-assemble in vitro and in vivo. *Nat. Biotechnol.* **35,** 1094–1101 (2017).

8. Krantz, B. A. & Sosnick, T. R. Distinguishing between two-state and three-state models for ubiquitin folding. *Biochemistry* **39,** 11696–11701 (2000).

9. Morrone, A., Giri, R., Brunori, M. & Gianni, S. Reassessing the folding of the KIX domain: Evidence for a two-state mechanism. *Protein Sci.* **21,** 1775–1779 (2012).

10. Briggs, M. S. & Roder, H. Early hydrogen-bonding events in the folding reaction of ubiquitin. *Proc. Natl. Acad. Sci. U. S. A.* **89,** 2017–2021 (1992).

11. Horng, J. C., Tracz, S. M., Lumb, K. J. & Raleigh, D. P. Slow folding of a three-helix protein via a compact intermediate. *Biochemistry* **44,** 627–634 (2005).

12. Zitzewitz, J. A., Ibarra-Molero, B., Fishel, D. R., Terry, K. L. & Robert Matthews, C. Preformed secondary structure drives the association reaction of GCN4-p1, a model coiled-coil system. *J. Mol. Biol.* **296,** 1105–1116 (2000).

13. Lacroix, E., Viguera, A. R. & Serrano, L. Elucidating the folding problem of alpha-helices: local motifs, long-range electrostatics, ionic-strength dependence and prediction of NMR parameters. *J. Mol. Biol.* **284,** 173–91 (1998).
