# Supplementary material for: Designed folding pathway of modular coiled-coil-based proteins
Source: Nat Commun. 2021 Feb 11;12:940. doi: 10.1038/s41467-021-21185-5 (PMC7878764; doi:10.1038/s41467-021-21185-5)
Supplement: Supplementary file 3 — Description of Additional Supplementary Files [file 41467_2021_21185_MOESM3_ESM.pdf]

Description of Additional Supplementary Files

File Name: Supplementary Data 1
Description: Python script for predicting the probability of folding for CCPO tetrahedra with repeating CC building blocks as described in the main text. The zip folder additionally contains a README file with instructions for use and one example output.

File Name: Supplementary Movie 1
Description: Representative molecular dynamics (MD) folding trajectory for TET12SN. The MD simulations were performed with the Gō force field. CC pairs are coloured as follows: APH – red, BCR – green, GCN – brown, P3:P4 – blue, P5:P6 – orange, P7:P8 – violet.

File Name: Supplementary Movie 2
Description: Representative molecular dynamics (MD) folding trajectory for TET12(2.3)SN-f5b. The MD simulations were performed with the Gō force field. CC pairs are coloured as follows: APH – red, BCR – green, APH4 – grey, P3:P4 – blue, P5:P6 – orange, P7:P8 – violet.

File Name: Supplementary Movie 3
Description: Representative molecular dynamics (MD) folding trajectory for TET12(1.11)S-f5. The MD simulations were performed with the Gō force field. CC pairs are coloured as follows: APH – red, BCR – green, GCN – brown, P3:P4 – blue, P5:P6 – orange, P7:P8 – violet.

File Name: Supplementary Movie 4
Description: Representative molecular dynamics (MD) folding trajectory for TET12(1.6)S-c6b. The MD simulations were performed with the Gō force field. CC pairs are coloured as follows: APH – red, BCR – green, GCN – brown, P3:P4 – blue, P5:P6 – orange, P7:P8 – violet.
