# Supplementary material for: Designed folding pathway of modular coiled-coil-based proteins
Source: Nat Commun. 2021 Feb 11;12:940. doi: 10.1038/s41467-021-21185-5 (PMC7878764; doi:10.1038/s41467-021-21185-5)
Supplement: Supplementary file 9 — Reporting Summary [file 41467_2021_21185_MOESM9_ESM.pdf]

nature research

Corresponding author(s): Roman Jerala

Last updated by author(s): Dec 17, 2020

Reporting Summary

Nature Research wishes to improve the reproducibility of the work that we publish. This form provides structure for consistency and transparency in reporting. For further information on Nature Research policies, see our Editorial Policies and the Editorial Policy Checklist.

Statistics

For all statistical analyses, confirm that the following items are present in the figure legend, table legend, main text, or Methods section.

n/a	Confirmed	
☐	☒	The exact sample size (*n*) for each experimental group/condition, given as a discrete number and unit of measurement
☐	☒	A statement on whether measurements were taken from distinct samples or whether the same sample was measured repeatedly
☒	☐	The statistical test(s) used AND whether they are one- or two-sided<br>*Only common tests should be described solely by name; describe more complex techniques in the Methods section.*
☒	☐	A description of all covariates tested
☒	☐	A description of any assumptions or corrections, such as tests of normality and adjustment for multiple comparisons
☐	☒	A full description of the statistical parameters including central tendency (e.g. means) or other basic estimates (e.g. regression coefficient) AND variation (e.g. standard deviation) or associated estimates of uncertainty (e.g. confidence intervals)
☒	☐	For null hypothesis testing, the test statistic (e.g. *F*, *t*, *r*) with confidence intervals, effect sizes, degrees of freedom and *P* value noted<br>*Give P values as exact values whenever suitable.*
☒	☐	For Bayesian analysis, information on the choice of priors and Markov chain Monte Carlo settings
☒	☐	For hierarchical and complex designs, identification of the appropriate level for tests and full reporting of outcomes
☒	☐	Estimates of effect sizes (e.g. Cohen's *d*, Pearson's *r*), indicating how they were calculated

Our web collection on statistics for biologists contains articles on many of the points above.

Software and code

Policy information about availability of computer code

Data collection	MD simulations were performed with Gromacs (5.0.2).<br>CD data were collected with the software Chirascan 4.5 (Applied Photophysics, UK).<br>SEC-MALS measurements were collected with Astra 7.0 software (Wyatt, CA USA).<br>SAXS data were integrated in the SASFLOW pipeline (version 3.0)<br>Stopped-flow data were collected with the Bio-Kine32 (version 4.72, BioLogic, France).<br>Fluorescence spectra were collected with Gen5 (version 1.10, BioTeK, VT USA).<br>Fluorescence thermal denaturation profiles were collected with LightCycler 480 (version 1.5.1.62, Roche Diagnostics, Germany).
Data analysis	The CoCoPOD software (version 0.0.1) was used to design and model CCPO proteins.<br>CD measurements were analysed with model-fitting software glox (version 0.2.) described by Drobnak et al. (ref. 47).<br>Analysis of SAXS curves and ab initio modelling were performed using the ATSAS suite (version 2.8.0) .<br>SAXS profiles were calculated from molecular models and compared to experimental data using Pepsi-SAXS (version 3.0.).<br>Ab initio models were evaluated with PyMOL Molecular Graphics System (version 2.2.3).<br>Python (version 3.5.4) in combination with the lmfit package (version 0.9.11) was used to analyse FRET measurements.

For manuscripts utilizing custom algorithms or software that are central to the research but not yet described in published literature, software must be made available to editors and reviewers. We strongly encourage code deposition in a community repository (e.g. GitHub). See the Nature Research guidelines for submitting code & software for further information.

Data

Policy information about availability of data

All manuscripts must include a data availability statement. This statement should provide the following information, where applicable:

- Accession codes, unique identifiers, or web links for publicly available datasets
- A list of figures that have associated raw data
- A description of any restrictions on data availability

SAXS data for TET12SN(2CC), TET12SN(22CC) and TET12SN(222CC) have been deposited to SASBDB (accession codes: SASDKQ2 [https://www.sasbdb.org/data/SASDKQ2/], SASDKR2 [https://www.sasbdb.org/data/SASDKR2/] and SASDKS2 [https://www.sasbdb.org/data/SASDKS2/], respectively). Source data are provided with this paper.

Field-specific reporting

Please select the one below that is the best fit for your research. If you are not sure, read the appropriate sections before making your selection.

☒ Life sciences ☐ Behavioural & social sciences ☐ Ecological, evolutionary & environmental sciences

For a reference copy of the document with all sections, see nature.com/documents/nr-reporting-summary-flat.pdf

Life sciences study design

All studies must disclose on these points even when the disclosure is negative.

Sample size	No sample-size calculations were used. Sample-sizes were chosen based on experimental experience with previously reported CCPO cages. Low-deviation between individual measurements suggests sample-sizes were sufficient.
Data exclusions	No data was excluded.
Replication	Reproducibility of SAXS data was confirmed by redundancy in the data collection. For each protein sample, scattering profiles were collected at four different concentrations. For each concentration, 40 scattering profiles were collected.<br>Biophysical analysis (stopped-flow, circular dichroism, FRET and fluorescence measurements) were repeated in triplicates on at least two independent samples and were reproducible. SEC-MALS measurements were performed on two independent samples and were reproducible.<br>SDS-PAGE experiments were performed at least three times for each protein with comparable outcomes.
Randomization	No randomization was performed, since in all experiments all samples were prepared, treated and analysed in the same manner.
Blinding	Not relevant for reported experiments, since data collection and method of analysis were not affected by the knowledge of sample identity.

Reporting for specific materials, systems and methods

We require information from authors about some types of materials, experimental systems and methods used in many studies. Here, indicate whether each material, system or method listed is relevant to your study. If you are not sure if a list item applies to your research, read the appropriate section before selecting a response.

Materials & experimental systems

n/a	Involved in the study	
☒	☐	Antibodies
☒	☐	Eukaryotic cell lines
☒	☐	Palaeontology and archaeology
☒	☐	Animals and other organisms
☒	☐	Human research participants
☒	☐	Clinical data
☒	☐	Dual use research of concern

Methods

n/a	Involved in the study	
☒	☐	ChIP-seq
☒	☐	Flow cytometry
☒	☐	MRI-based neuroimaging
